# Notch pathway mutants do not equivalently perturb mouse embryonic retinal development

**DOI:** 10.1371/journal.pgen.1010928

**Published:** 2023-09-26

**Authors:** Bernadett Bosze, Julissa Suarez-Navarro, Illiana Cajias, Joseph A. Brzezinski IV, Nadean L. Brown

**Affiliations:** 1 Department of Cell Biology & Human Anatomy, University of California, Davis, California, United States of America; 2 Department of Ophthalmology, University of Colorado Anschutz Medical Campus, Aurora, Colorado, United States of America; Johns Hopkins University School of Medicine, UNITED STATES

## Abstract

In the vertebrate eye, Notch ligands, receptors, and ternary complex components determine the destiny of retinal progenitor cells in part by regulating *Hes* effector gene activity. There are multiple paralogues for nearly every node in this pathway, which results in numerous instances of redundancy and compensation during development. To dissect such complexity at the earliest stages of eye development, we used seven germline or conditional mutant mice and two spatiotemporally distinct Cre drivers. We perturbed the Notch ternary complex and multiple *Hes* genes to understand if Notch regulates optic stalk/nerve head development; and to test intracellular pathway components for their Notch-dependent versus -independent roles during retinal ganglion cell and cone photoreceptor competence and fate acquisition. We confirmed that disrupting Notch signaling universally blocks progenitor cell growth, but delineated specific pathway components that can act independently, such as sustained Hes1 expression in the optic stalk/nerve head. In retinal progenitor cells, we found that among the genes tested, they do not uniformly suppress retinal ganglion cell or cone differentiation; which is not due differences in developmental timing. We discovered that shifts in the earliest cell fates correlate with expression changes for the early photoreceptor factor Otx2, but not with Atoh7, a factor required for retinal ganglion cell formation. During photoreceptor genesis we also better defined multiple and simultaneous activities for *Rbpj* and *Hes1* and identify redundant activities that occur downstream of Notch. Given its unique roles at the retina-optic stalk boundary and cone photoreceptor genesis, our data suggest Hes1 as a hub where Notch-dependent and -independent inputs converge.

## Author summary

A long-standing question in biology is how cells respond to multiple signaling inputs with a specific response. Here we directly compared the genetic requirements of multiple genes in the Notch signaling pathway. These genes are components of a molecular cascade in responding cells that is triggered by Notch receptors binding to ligands on an adjacent cell. Notch signaling is an important regulator of retinal neuron formation, which acts in the presence of other signals. This results in multiple pathways converging on key, shared downstream target genes. Here we genetically removed four different Notch pathway genes during mouse embryonic eye development, either alone or in combinations, and analyzed the consequences. We found three situations, during tissue specification and retinal neurogenesis where the activities of these genes have both Notch-dependent and Notch-independent activities. Our data significantly extend current models of how the retina distinguishes itself from other tissues and how retinal progenitor cells decide to stop dividing and select particular neuronal fates. These findings extend current models regarding integration or branchpoints for signaling cascades.

## Introduction

The central eye field in vertebrate embryos is specified at the end of gastrulation and splits to form bilateral optic vesicles that evaginate from the ventral diencephalon. Multiple signaling pathways regionalize and pattern the growing optic vesicles, demarcating the optic stalk (OS), optic cup (OC) and retinal pigment epithelium (RPE) tissues. The OC gives rise to the neural retina, which is an excellent system for studying cell fate specification and differentiation. The retina is comprised of seven major cell classes that arise in a tightly controlled, but overlapping chronological order: retinal ganglion cells (RGCs), cone photoreceptors, horizontals, and a subset of amacrine neurons—before birth; and amacrines, rods, bipolars and Müller glia—mainly after birth. These cell types are derived from proliferative multipotent retinal progenitor cells (RPC) that permanently stop diving before differentiating into neurons and glia. Throughout development, RPC pool size must be balanced with neuron and glia production to generate a functional retina [reviewed in 1,2].

The highly conserved Delta-Notch signaling pathway maintains the equilibrium between proliferation and differentiation in a myriad of tissues and often acts reiteratively within a single organ [3,4]. In brief, signaling starts at the cell membrane upon ligand-receptor binding, which induces sequential proteolytic cleavages of the Notch receptor and ultimately releases the Notch intracellular domain (N-ICD). N-ICD forms a ternary complex with Rbpj (Recombination signaling binding protein, also termed CBF1) and Maml (Mastermind-like) [4]. These ternary complexes bind DNA to transcriptionally activate target genes, including *Drosophila* ***H****airy* or ***E(s****pl)*, and vertebrate *Hes* gene families [5–7]. In several tissues, the loss of canonical Notch signaling results in precocious flawed neurogenesis, whereas too much signaling induces overproliferation [8–21]. Therefore, the Notch pathway controls the balance between proliferation and differentiation during retinal development. Throughout development, naive RPCs progress through a transitional state, exit mitosis, commit to a fate, and differentiate [reviewed in 22]. Transitional RPCs downregulate the Notch reception machinery, but upregulate Notch ligands, presumably to communicate with nearby, naive RPCs [23–25]. Transitional RPCs also turn on competence factors that are necessary for neuronal fate choice, such as *Atoh7* for RGCs [26–31], and *Otx2* for photoreceptors [32–34]. The mechanisms for how competence factors steer cells to distinct cell fates, and their dependence on Notch signaling, remain unresolved.

Most vertebrate *Hes* genes are Notch ternary complex targets [7,35–38]. *Hes1*, *3* and *5* are important in the nervous system, whereas *Hes2*, *4* and *7* act in other parts of the body [7,39]. The role of *Hes6* in development is debatable [reviewed in 7]. Both *Hes1* and *Hes5* can exhibit oscillating expression patterns within stem cells or neural progenitors poised between proliferation and differentiation [39]. For example, actively proliferating progenitor cells show high, oscillating *Hes1* levels, whereas low *Hes1* correlates with differentiation [40]. In the mouse spinal cord, *Hes5* can be either sustained or oscillatory, with its frequency of oscillation correlating with onset of differentiation [41]. *Hes1* is an essential gene, whose loss causes prenatal lethality along with embryonic morphogenesis defects characterized by premature differentiation [42]. By comparison, complete loss of *Hes3* and/or *Hes5* has no impact on viability, but can induce discrete defects, suggesting specific contexts when these paralogues are compensated by or redundant with *Hes1*. This is further supported by the increased severity of *Hes1;Hes3;Hes5* triple mutants in other parts of the central nervous system (CNS) [43–48]. Despite the importance of the Notch pathway in retinal neurogenesis, no functions have been reported for it during mammalian optic vesicle/cup outgrowth, patterning or morphogenesis. Moreover, *Hes* gene redundancy and compensation have not been explored in the developing retina or adjacent tissues. In the E13.5 mouse eye, both *Hes1* expression modes are present. RPCs oscillate while adjacent ONH/OS cells exhibit sustained *Hes1* expression [49,50]. As a Notch ternary complex target, removing *Hes1* is predicted to universally release the block on neuron differentiation, but paradoxically *Hes1* retinal mutants simultaneously have excess RGC neurons, but too few cone photoreceptors [14,42,50,51] (S1 Table). This implies the *Hes1* gene is where Notch-independent [52] and Notch-dependent regulation converge, with the latter complicated by *Hes* gene redundancy or compensation.

To understand the complexity of *Hes* gene function during development, we directly compared the embryonic eye phenotypes of *Hes* single versus multiple mutant mice [43,53]. Because *Hes* triple germline mutants die soon after gastrulation, a *Hes1* conditional mutation (*Hes1*^*CKO/CKO*^) was combined with *Hes3*^*-/-*^*;Hes5*^*-/-*^ germline mutant alleles, to effectively generate tissue-specific *Hes* triple mutants (*Hes*^*TKO*^) [43,53]. Importantly, we also asked how well *Hes*^*TKO*^ phenotypes match the rest of the Notch pathway by evaluating *Rbpj*^*CKO/CKO*^ and *ROSA*^*dnMaml-GFP/+*^ retinal mutants. The *ROSA*^*dnMaml-GFP/+*^ allele is under flox-stop control, and dominantly creates inactive Notch transcriptional complexes, using a truncated Mastermind-nGFP fusion protein that binds with endogenous N-ICD and Rbpj [54–57]. For this study we used two Cre drivers (Rax-Cre and Chx10-Cre) with spatially overlapping, but temporally offset Cre activation, to tease apart morphologic versus neurogenic roles for each gene [50,58]. These experiments facilitated a direct phenotypic comparison among the allelic series, and integration of our findings with those from previous studies (S1 Table) [8–17].

Our direct comparisons of *Hes*^*TKO*^ versus *Rbpj* conditional mutants support that *Hes* genes regulate the balance between RPC growth and neurogenesis progression. We also discovered that Maml cofactor activities are not exclusive to the Notch ternary complex, in that *ROSA*^*dnMaml-GFP/+*^ retinal mutants have unique nasal-temporal patterning defects. We determined that sustained Hes1 expression is Notch-independent, whereas in the retinal compartment, *Hes1* and *Hes5* are partially redundant downstream of Notch. Our phenotypic analyses of early neurogenesis reveal both Notch-dependent and -independent functions that influence RPC progression into early competence states, and further highlight directly opposing roles for *Rbpj* and *Hes1* regarding cone fate. Although *Hes*^*TKO*^ mutants partially rescue the *Hes1* cone phenotype, they do not fully recapitulate those of *Notch1* or *Rbpj* mutants [10,14,16,17]. We conclude that unknown genetic inputs, independent from Notch signaling, also impact early neurogenesis and act via competence factors to affect RGC and cone photoreceptor fate determination.

## Results

**Fig 1:**
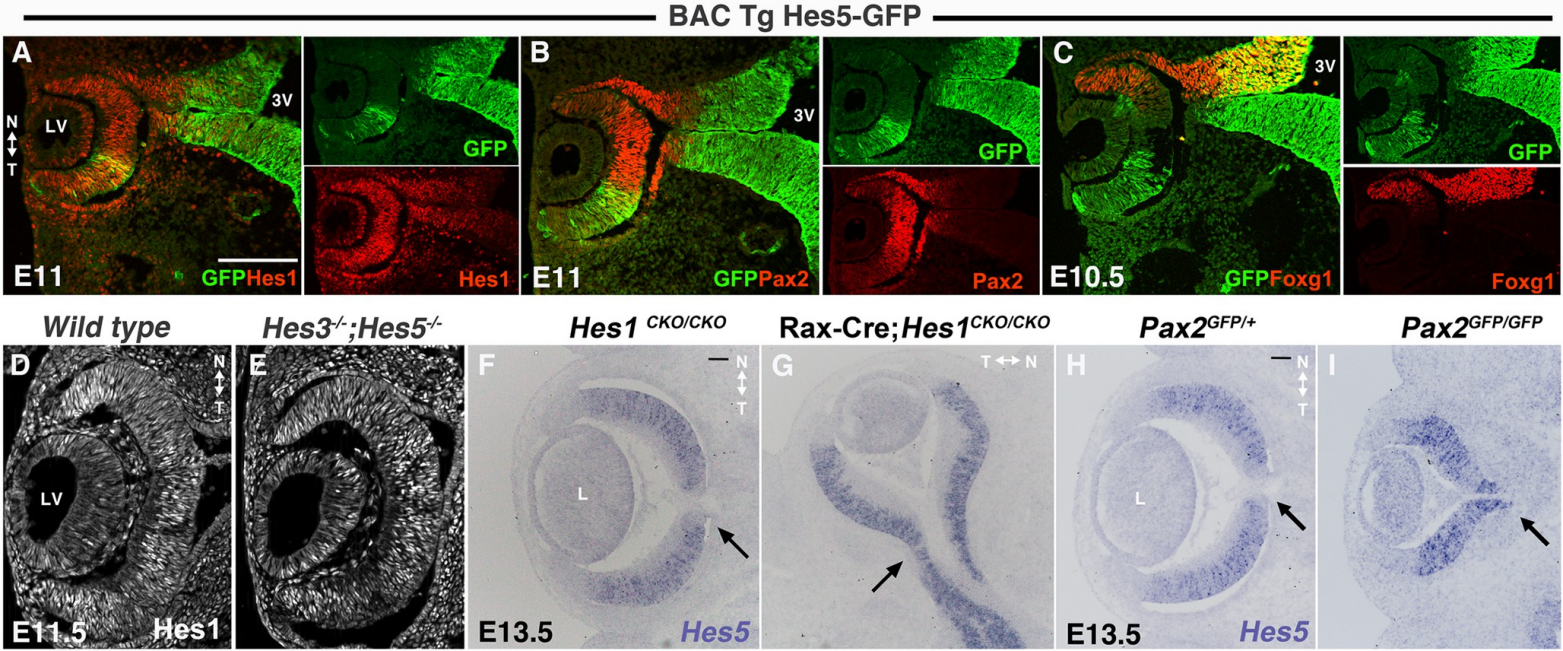
*Hes* genes in the embryonic mouse eye. **(**A) Hes1 and Hes5-GFP colabeling at E11.0 shows uniform Hes1 expression, and BAC Tg(*Hes5*-GFP) expression in the temporal optic cup and most of the optic stalk and adjacent diencephalon. (B) At this stage, Pax2 and Hes5-GFP are largely mutually exclusive, with Pax2 expression transiting from uniform to ONH/OS domain restriction [65]. OS and brain progenitors surrounding the third ventricle (3V) have not yet differentiated. (C) At E10.5 Hes5-GFP and Foxg1 are not coexpressed in the optic cup, as they are in the nasal brain (yellow domain). (D,E) By E11.5, Hes1 now exhibits oscillating optic cup expression, which is unaffected in *Hes3*^*-/-*^*;Hes5*^*-/-*^ double mutants. (F-G) At E13.5 *Hes5* mRNA expression is inappropriately expanded in the retinal territory that invaded the OS (arrows), after Rax-Cre removal of *Hes1*(G); Chx10-Cre-induced *Hes1* mutants have normal *Hes5* expression (S2 Fig). (H-I) *Hes5* mRNA similarly expands in *Pax2*^*GFP/GFP*^ mutants with retina-ONH boundary (arrows) defects [65]. N = nasal; T = temporal; LV = lens vesicle; L = lens; 3V = third ventricle; Bar in A = 10 microns, in F,H = 100 microns; n ≥3 per age and genotype.

**Fig 2:**
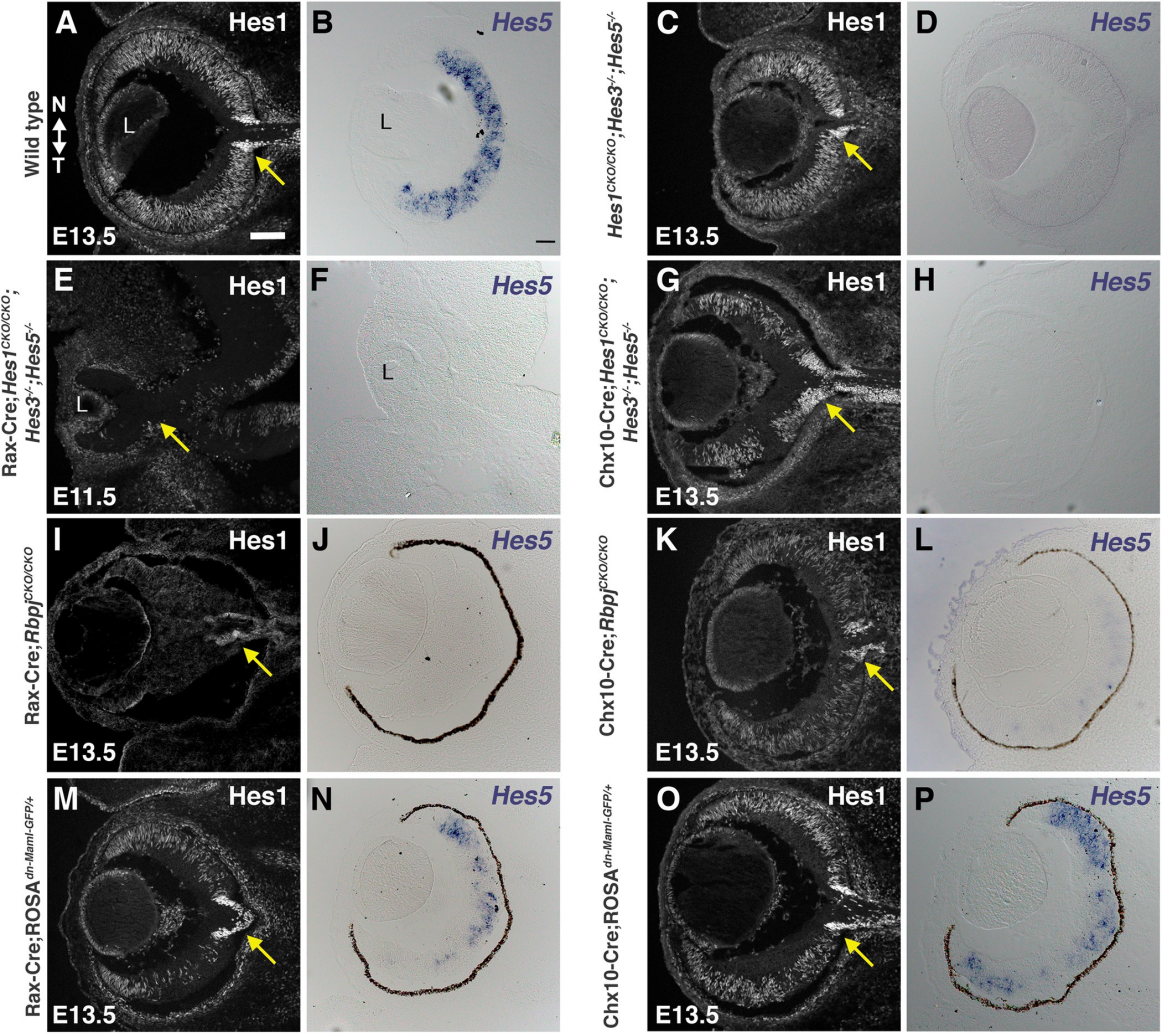
*Hes1* and *Hes5* expression in *Rbpj*, dnMAML and *Hes* triple retinal mutants. (A,C,E,G,I,K,M,O) Anti-Hes1 labeling of E11.5 or E13.5 cryosections. Hes1 is missing in Rax-Cre;*Hes*^*TKO*^ and Rax-Cre;*Rbpj*^*CKO/CKO*^ RPCs (E, I), with the intense Hes1+ ONH domain (yellow arrows) only lost in Rax-Cre;*Hes*^*TKO*^ eyes. (B,D,F,H,J,L,N,P) *Hes5* mRNA is missing in all *Hes5* germline mutants. Both *Rbpj* conditional mutants effectively block *Hes5* mRNA expression (J,L). dnMAML partially knocks down Hes1 (M,O) and *Hes5* (N,P). The effect is stronger in Rax-Cre;*ROSA*^*dnMaml1-GFP/+*^ eyes, but both conditions showing a more pronounced effect in the temporal optic cup. All panels oriented nasal up and temporal down (noted in A), with L = lens in A,B,E,F; scalebar in A = 100 microns, B = 50 microns; n = 3/3 mutants per genotype.

During mouse nervous system development, *Hes1* appears in the anterior neural plate, optic vesicle and optic cup several days prior to the onset of retinal neurogenesis [42,59]. We first compared the expression of multiple *Hes* genes throughout embryonic eye development (Fig 1). At these early stages, *Hes1* mRNA and protein are uniformly expressed (Fig 1A). As the first cohort of retinal progenitor cells (RPCs) cells exit mitosis and differentiate into neurons, there is a switch in Hes1 expression to a "salt-n-pepper" pattern within mitotic RPCs (Fig 1D). However, optic nerve head (ONH) and optic stalk (OS) cells retain uniform Hes1 expression [50] (also see Fig 2A). By contrast, *Hes5* mRNA appears later within RPCs just ahead of the first neurons [60]. The mouse Hes5-GFP BAC transgene is an accurate reporter of *Hes5* expression, enabling direct correlation with Hes1 and other markers during development [60]. Hes5-GFP is also found in the diencephalon (Fig 1A–1C), but not in optic stalk cells that express Pax2 (Fig 1B). At E11, there are no Hes5-GFP+ cells in the nasal optic cup as marked by Pax2 and Foxg1 (Fig 1B and 1C). *Hes3* functionally overlaps with *Hes1* in the brain isthmus and is active in the CNS as early as E9.5 [61]. Nonetheless, we did not detect *Hes3* mRNA in the retina prior to E18 [62]. We conclude that *Hes1* is activated well before *Hes5*, which turns on in a subset of RPCs just prior to the onset of neurogenesis. *Hes1* is expressed in distinct modes, appearing to oscillate in RPCs while exhibiting a high sustained level in the optic stalk.

Next, we asked whether Hes1 depends on other *Hes* genes. *Hes3* and *Hes5* are <1Mb apart on mouse chromosome 4, and their knockout alleles are transmitted together as one mutant haplotype (S2 Table) [43,49]. We confirmed that *Hes3*^*-/-*^*;Hes5*^*-/-*^ mutants have normal retinal morphology and cell-type composition across eight developmental stages (E10.5-P21) (S1 Fig). We examined Hes1 ocular expression from E10.5-E16.5 within *Hes3*^*-/-*^*;Hes5*^*-/-*^ mice, and found that both oscillating RPC and sustained ONH/OS Hes1 domains were normal (Figs 1D,1E and 2C). Thus, *Hes1* is not cross-regulated by either *Hes3* or *Hes5*. Then, we checked for reciprocal regulation by evaluating *Hes5* mRNA in E13.5 and E16.5 *Hes1* conditional (CKO) mutants, using two Cre drivers whose activation is temporally offset. Rax-Cre initiates recombination as early as E8.5 and acts in the ventral thalamus/hypothalamus, optic vesicle, cup and stalk, RPE, ONH and RPCs. The Chx10-Cre driver deletes genes from E10.5 onwards, exclusively in RPCs [50,58,63]. Upon earlier and broader deletion using Rax-Cre, *Hes5* mRNA abnormally extends into the E13.5 optic stalk (Fig 1G), whereas *Hes5* mRNA was unaffected in later-deleting Chx10-Cre;*Hes1*^*CKO/CKO*^ mutant retinas (S2 Fig). Previous studies suggested that Hes1 can suppress *Hes5* in the developing CNS [46,60,64]. However, expansion of the *Hes5* mRNA domain in Rax-Cre;*Hes1*^*CKO/CKO*^ retinas could be coincident with ectopic retinal tissue formation in this mutant [38]. To distinguish between these possibilities, we assayed *Hes5* expression in *Pax2*^*GFP/GFP*^ (germline) mutants, which also have ectopic retinal tissue in the optic stalk [65,66]. Here too, we found the *Hes5* mRNA domain was inappropriately expanded (Fig 1I). Thus, we conclude that ectopic retina formation, rather than *Hes1* suppression of *Hes5*, is the cause of expanded *Hes5* in our Rax-Cre;*Hes1*^*CKO/CKO*^ mutants.

**Fig 3:**
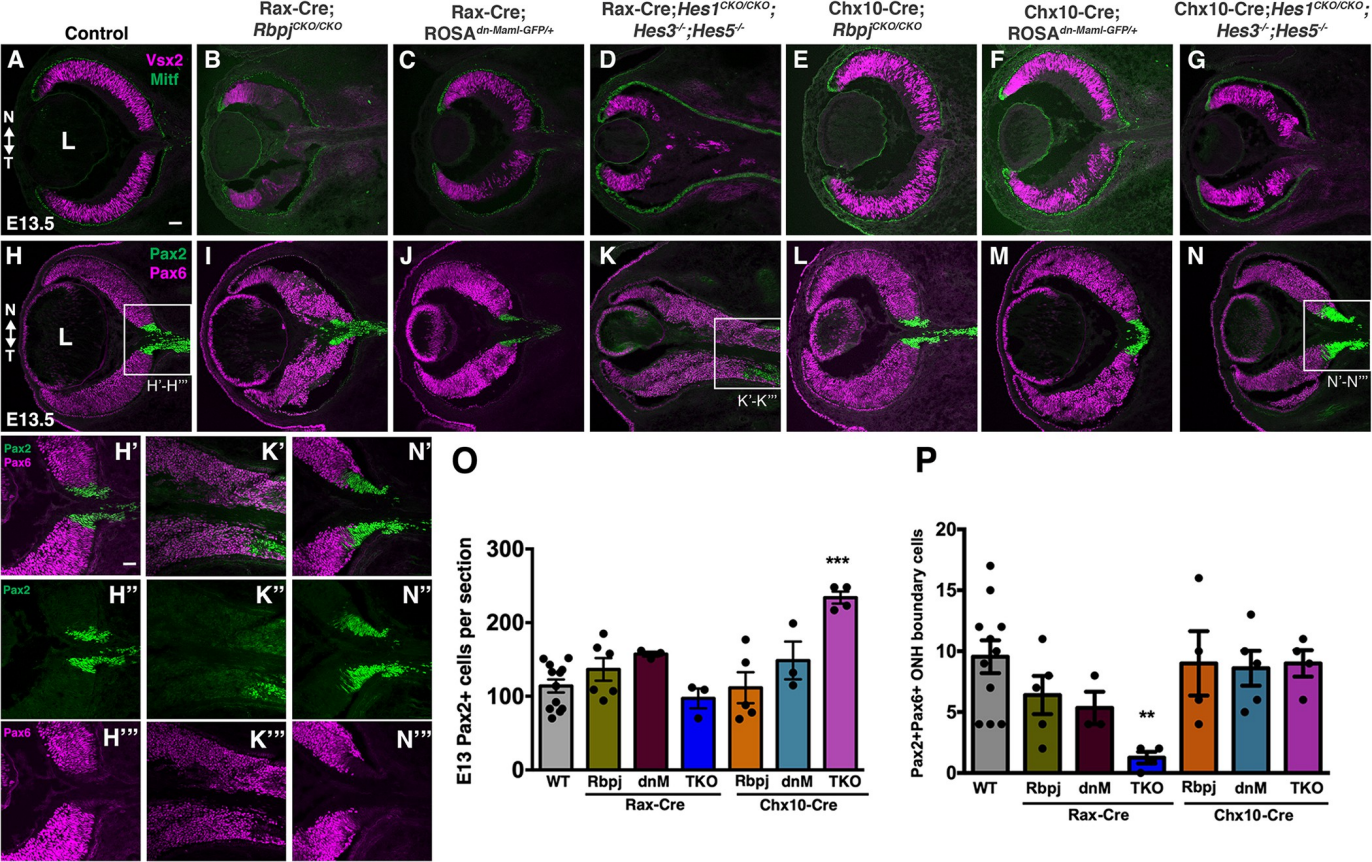
Ocular tissue patterning defects among Notch pathway mutants. (A-G) Vsx2 and Mitf double labeling marks E13.5 RPCs (fuchsia) and RPE cells (green), respectively. Vsx2+ RPCs were disorganized in the all mutants, with this domain displaced in Rax-Cre;*Rbpj*^*CKO/CKO*^ and Rax-Cre;*Hes*^*TKO*^ eyes (B,D). (H-N) Pax6-Pax2 colabeling delineates the retinal-optic stalk boundary, with boxed regions in H,K,N shown at higher magnification in H’ to N”’. (O) Quantification of total Pax2+ cells per section (P) Quantification of Pax6+Pax2+ cells per section at retinal-ONH interface (both boundaries). Graphs display individual replicate data points, the mean and S.E.M; Significant Welch’s ANOVA, plus pairwise comparisons to wild type (***p<0.001, **p<0.01). Rax-Cre;*Hes*^*TKO*^ eyes have a more elongated Pax6 domain (K, K’-K”’) with no impact on the size of the Pax2+ domain (O), but a significant loss of double-labeled boundary cells (P). Only Chx10-Cre;*Hes*^*TKO*^ eyes had an enlarged Pax2 domain (N, N’-N”’;O). All panels oriented nasal up (noted in A, H), L = lens in A,H; scalebar = 50 microns in A, 100 microns in H’; n ≥ 3 biologic replicates/genotype.

**Fig 4:**
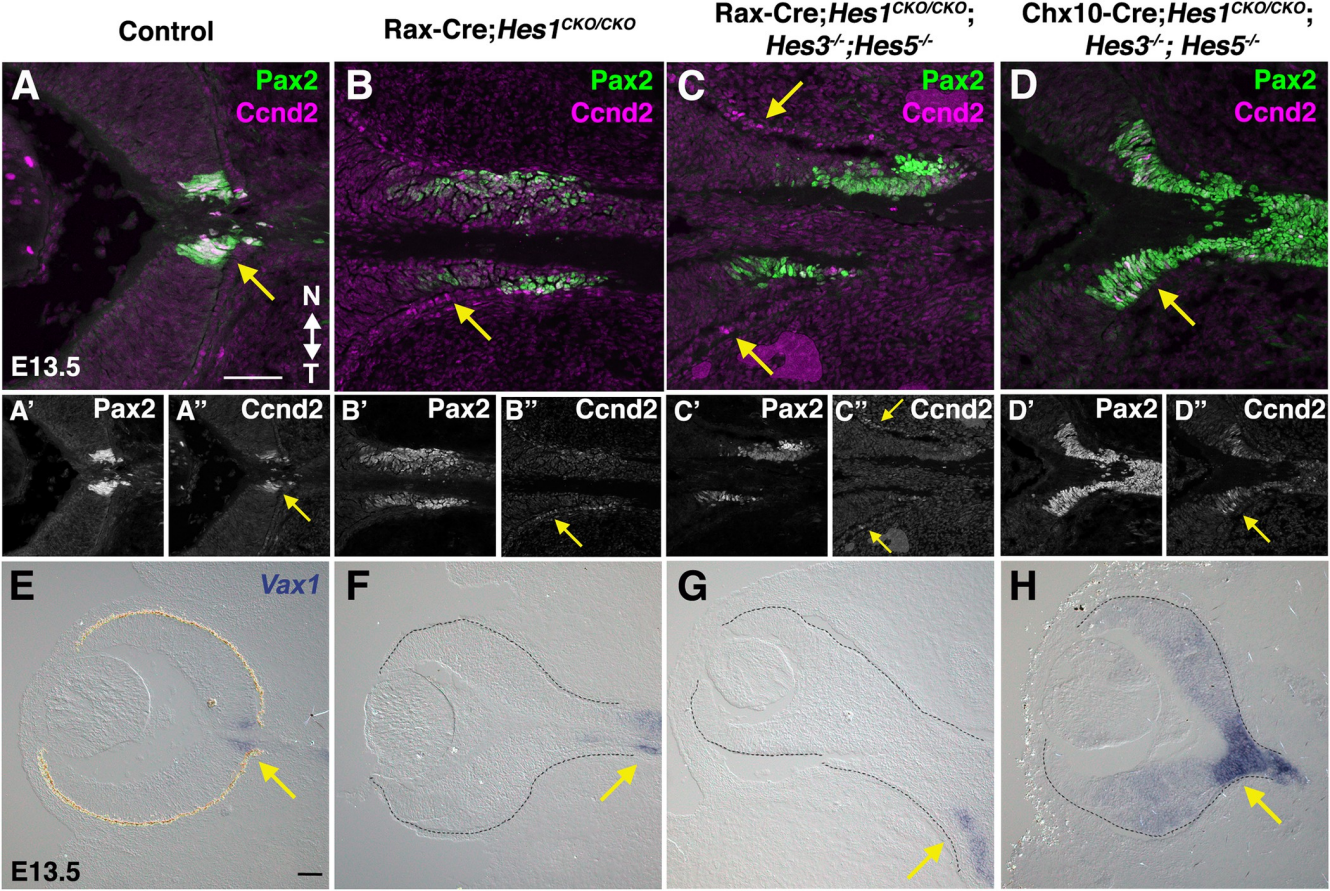
*Hes1 and Hes*^*TKO*^ retina-ONH boundary phenotypes. (A-D") Pax2 and Ccnd2 immunolabeling at E13.5. Normally, Pax2 and Ccnd2 are coexpressed in ONH cells. In Rax-Cre;*Hes1*^*CKO/CKO*^ and Rax-Cre;*Hes*^*TKO*^ eyes, the Pax2 OS domain is elongated, with Ccnd2 expression dramatically downregulated in the optic stalk or mislocalized into the RPE (arrows in A,A", B,B",C,C"). Intriguingly, in Chx10-Cre;*Hes*^*TKO*^ eyes, both Pax2 and Ccnd2 domains expanded into the optic cup (arrows in D, D”). (E-H) *Vax1* mRNA expression in the ONH/OS (arrows). Eyes in F-H are albino and the retina is outlined with dotted lines. The *Vax1* domain shifted toward the brain in Rax-Cre;*Hes1*^*CKO/CKO*^ and Rax-Cre;*Hes*^*TKO*^ eyes, but in Chx10-Cre;*Hes*^*TKO*^ eyes it was expanded both into the retina and towards the brain. All panels oriented nasal up (noted in A) and the diencephalon to the right; n = 3 biologic replicates/genotype.

The loss of multiple *Hes* genes is more catastrophic than loss of *Hes1* alone in several regions of the embryo [reviewed in 7]. We used the two Cre drivers with *Hes*^*TKO*^ mice (*Hes1*^*CKO/CKO*^*; Hes3*^*-/-*^*;Hes5*^*-/-*)^ to test this idea in the optic cup and stalk. We collected litters at E11, E13.5, E16.5, P0 (birth) (S2 Table). Rax-Cre;*Hes*^*TKO*^ mutants were not viable beyond E13, but displayed more severe phenotypes than *Hes1* single mutants (S2 Table, Figs 3 and 4) [50]. For the surviving Chx10-Cre;*Hes*^*TKO*^ mice, we directly compared their P21 ocular phenotypes to Chx10-Cre;*Hes1*^*CKO/CKO*^ single mutants (S3 Fig). *Hes1* single mutants had defective retinal lamination, rosettes, and occasionally a small, vitreal cell mass (S3B Fig boxed area). By contrast, adult Chx10-Cre;*Hes*^*TKO*^ eyes had more severe retinal lamination and rosetting defects and conspicuous microphthalmia (S3C and S3D Fig). In some sections, ectopic tissue in the vitreous appeared contiguous with the ONH (S3C and S3D Fig boxed areas). We performed Tubb3/Endomucin (Emcn) colabeling of the ectopic tissue to assay for neurons and blood vessels, respectively (S3E–S3L’ Fig). Although blood vessels (Emcn+ cell membranes, pink arrows) and autofluorescent red blood cells (asterisks) were obvious, Tubb3+ neurons were difficult to observe, suggesting this ectopic tissue may have a nonneuronal origin. Overall, we observed that *Hes*^*TKO*^ mutants are more severe than single *Hes1* mutants or *Hes3/5* double mutants. Our findings argue that *Hes* genes act in a complex, yet incompletely redundant fashion during eye development. To unravel this complexity, we initiated a deeper phenotypic evaluation at E13.5, when Rax-Cre triple mutants are viable and the ONH is fully formed.

### *Hes*^*TKO*^ and *Rbpj* mutants are the most severe

In theory, combined *Hes* functions should reflect those of the Notch ternary complex, which transcriptionally activates *Hes* genes. So we asked to what extent *Hes*^*TKO*^ ocular mutants phenocopy the loss of ternary complex gene function. This also allowed us to bypass complexity at the receptor level, as three Notch receptors are expressed in the prenatal mouse eye [11,67]. We opted to directly compare conditional mutant phenotypes for *Rbpj* and *dnMAML* (dominant allele that creates inactive Notch transcriptional complexes) to those for *Hes*^*TKO*^, using the same Cre drivers (Fig 2). In E11.5 Rax-Cre;*Hes*^*TKO*^ eyes, RPC and ONH/OS cells are devoid of Hes1 protein and *Hes5* mRNA as expected (Fig 2E). Because Chx10-Cre activates later and only within the retina [50], we expected there would be a loss of Hes1 from RPCs, but not ONH/OS cells. However, in E13.5 Chx10-Cre;*Hes*^*TKO*^ eyes, Hes1 clearly persists in both domains (compare Fig 2A and 2G). Since Hes1 is spotty in the retina and dependent upon Cre mediated recombination, we hypothesized this its pattern is due to mosaic Chx10-Cre expression [58,68]. This is further supported by immunostaining for Rbpj in Rax-Cre versus Chx10-Cre *Rbpj*^*CKO/CKO*^ mutants (S4 Fig). Moreover, we observed that E13.5 Rax-Cre;*Rbpj*^*CKO/CKO*^ mutants had a cell autonomous loss of Rbpj from RPC, ONH/OS, and RPE cells as expected (compare S4A, S4B and S4B’ Fig). Although Hes1 was absent from the optic cup and RPE (compare S4A’ and S4B" Fig), ONH/OS cells still express Hes1. Thus, we conclude sustained Hes1 expression in the ONH/OS is independent of Notch, whereas its expression in the retina depends upon Rbpj and Notch signaling.

We took advantage of a Cre-GFP fusion protein within the Chx10-Cre driver to directly compare GFP and Rbpj coexpression in Chx10-Cre;*Rbpj*^*CKO/CKO*^ and control Chx10-Cre;*Rbpj*^*CKO/+*^ retinal sections (S4C, S4E, S4G and S4I Fig). This Chx10-Cre BAC transgene encodes a Cre-GFP fusion protein, allowing us to test cell autonomy in the GFP+ cell population [58]. At E13.5 we noted a strong autonomous knockdown of Rbpj protein (S4C" Fig vs S4E" Fig), yet at E16.5 there were more Rbpj-expressing retinal cells that lacked GFP, identifying them as wild type (compare S4G’ Fig to S4I’ Fig). Hes1 was partially autonomously downregulated at both ages, mirroring what was seen with Rbpj (S4D, S4F, S4H, S4J Fig). Thus, we concluded that Chx10-Cre phenotypes generated through E13.5 are informative, but beyond this stage the wild type cohort (GFP-neg) outcompetes mutant (GFP+) cells [69,70], providing ample levels of Notch signaling and partially rescuing development. Evaluation of *Hes5* mRNA further confirmed Rax-Cre as the more effective driver, since we could still detect *Hes5* in Chx10-Cre;*Rbpj*^*CKO/CKO*^ retinas (compare Fig 2J to Fig 2L). So, subsequent analyses were confined to E13.5, when Rax-Cre mutants are viable and Chx10-Cre mosaicism is less impactful. Next, we examined *Hes1* and *Hes5* expression in E13.5 Rax-Cre;*ROSA*^*dnMAML-GFP/+*^ and Chx10-Cre;*ROSA*^*dnMAML-GFP/+*^ retinas. Hes1 and *Hes5* are only modestly reduced, with a stronger effect seen in the temporal retina (Fig 2M–2P). There was a stronger knockdown in the Rax-Cre;*ROSA*^*dnMAML-GFP/+*^ retinas (Fig 2M–2P). In neither case did we observe a loss of Hes1 in the ONH/OS area, further suggesting that it is independent of Notch signaling. The moderate phenotypes we observed did not fit our expectation that dnMAML misexpression would closely match the loss of *Rbpj*. Thus, we presume this *dnMAML* allele exhibits only a partial dominant negative effect in the developing eye. We decided to analyze this allele further to learn when, where and the degree to which it mimics *Rbpj*^*CKO/CKO*^ and *Hes*^*TKO*^ mutants.

### Notch signaling has no impact optic cup patterning

The optic vesicle and cup are patterned along dorsal-ventral (D/V) and nasal-temporal (N/T) axes. *Hes1* mutants have no D/V ocular phenotypes [50,59]. We checked for mispatterning of the N/T axis, since the Pax2 domain is displaced in Rax-Cre; *Hes1*^*CKO/CKO*^ eyes, and *Pax2* germline mutants have abnormal N/T ocular patterning [65]. We compared the nasal-restricted marker Foxg1 [71,72] among the six Rax-Cre or Chx10-Cre-induced mutants at E13.5 and E16.5 (S5 Fig). We noted normal Foxg1 retinal expression, with two exceptions. At E13.5 Rax-Cre;*Hes*^*TKO*^ eyes, the Foxg1 nasal retinal domain was contiguous with the nasal optic stalk (S5D Fig). This is reminiscent of younger stages (Fig 1C), since at E13.5 Foxg1 in the wild type condition is no longer made in the nasal OS domain (S5A Fig). Based on RPC domain expansion into the optic stalk (Fig 1G, see below), we conclude that this change in Foxg1 expression is another indication that the retina has expanded. The other exception is in E16.5 Rax-Cre;*ROSA*^*dnMAML-GFP/+*^ mutants. In this case, Foxg1 was mislocalized to the temporal retina and subretinal space (arrow in S5J Fig), a cell-free zone between the apical retina and RPE. We presume these displaced cells are RPCs, since some Notch pathway mutants lose the outer limiting membrane along the apical side of the optic cup, allowing cells to spill into the subretinal space [73,74].

The optic cup splits into retina and RPE during or soon after DV/NT patterning of the retina. Vsx2/Chx10 (RPCs) and Mitf (RPE) transcription factors delineate these tissues, and actively maintain this boundary [75–77]. We compared Vsx2 and Mitf expression among all six E13.5 mutants (Fig 3A–3G), expecting a normal boundary, but that there would be fewer RPCs. All E13.5 Rax-Cre-generated mutants had noticeably smaller eyes (Fig 3B, 3C and 3D), but Chx10-Cre generated mutants were typically of normal size (Fig 3E–3G). For all six allelic combinations, the RPE formed correctly, but in Rax-Cre;*Hes*^*TKO*^ eyes this tissue extended into the optic stalk (Fig 3D), phenocopying Rax-Cre; *Hes1*^*CKO/CKO*^ mutants [50]. Rax-Cre;*Rbpj*^*CKO/CKO*^ and Rax-Cre;*Hes*^*TKO*^ mutants shared a RPC defect, namely patches of Vsx2-negative cells in the proximal optic cup, where neurogenesis normally initiates (Fig 3B–3D). Taken together, we conclude that Notch signaling has no overt role in D/V and N/T patterning, or retinal/RPE specification (Fig 3B).

### *Hes1* is Notch-independent at the optic cup-stalk boundary

At E12, the neural retina and optic stalk tissues become delineated, also establishing a ring of cells called the optic nerve head (ONH). ONH cells ultimately adopt glial fates and its interface with the retina is delineated by the generally abutting expression of the transcription factors Pax6 (RPCs) and Pax2 (ONH/OS) [66]. Although the molecular mechanisms regulating this boundary are not well understood, its formation requires both *Hes1* and *Pax2* activities [50,65,66]. To understand whether Notch signaling controls formation of this boundary, we performed Pax6/Pax2 colabeling at E13.5 among all mutants (Fig 3I–3N). The Rax-Cre;*Hes*^*TKO*^ eyes, were the most severe, with Pax6+ retinal tissue extending into the optic stalk territory, displacing the Pax2 domain (boxed area in Fig 3K). Although the Pax6-Pax2 boundary is intact in Rax-Cre;*Rbpj*^*CKO/CKO*^ eyes, the shape of the ONH was attenuated compared to controls (Fig 3I). Interestingly, the proximal-most optic cup cells, those lacking Vsx2, still expressed Pax6 (compare Fig 3B to Fig 3I), suggesting these cells may have differentiated into neurons, since Pax6 is also expressed by nascent RGCs [69,78]. The Rax-Cre;*ROSA*^*dnMAML-GFP/+*^ eyes were largely unaffected, but ONH shape was abnormal (Fig 3J). In all three Chx10-Cre generated mutants, a Pax6-Pax2 boundary was clearly discernable (Fig 3L–3N). But for Chx10-Cre;*Hes*^*TKO*^ mutants, Pax2 was uniquely ectopic within the retinal territory (box in Fig 3N), demonstrating overlapping *Hes* gene function at this boundary (Fig 3D, 3K and 3N). We quantified the total number of Pax2+ cells per section (Fig 3O) and the small number of Pax6-Pax2 coexpressing "boundary" cells (Fig 3P). These data confirmed that although displaced, the Pax2-expressing ONH is of typical size in Rax-Cre;*Hes*^*TKO*^ eyes (Fig 3K, 3K’, 3K"–3O), and there was a significant loss of Pax6-Pax2 coexpressing boundary cells, most likely due to retinal extension (Pax6-only cell, Fig 3P). Moreover, only Chx10-Cre;*Hes*^*TKO*^ mutant eyes had an expanded Pax2 domain (Fig 3N, 3N’–3N" and 3O), but with normal boundary cell composition (Fig 3P).

The ONH and brain isthmus share multiple features, including Pax2 and sustained Hes1 expression [49,61,79], Brain isthmus cells have slower cell cycle dynamics than those in adjacent neural compartments with oscillatory expression [80]. Cyclin D2 (Ccnd2) is expressed by brain glial cells and intermediate neural progenitors with slow cycling kinetics [81,82] and interestingly, E13.5 ONH cells normally express Ccnd2, which is regulated by Notch signaling in other ocular tissues [83,84]. We observed that Ccnd2 is downregulated in Rax-Cre;*Hes1*^*CKO/CKO*^ and Rax-Cre;*Hes*^*TKO*^ mutants with mispositioned Pax2 domains (arrows in Fig 4B and 4C). Interestingly, Chx10-Cre;*Hes*^*TKO*^ eyes also downregulate Ccnd2 expression. Because *Hes1* encodes a transcriptional repressor, we presume its impact on Ccnd2 expression to be indirect. Once again, only Chx10-Cre;*Hes*^*TKO*^ retinal cells ectopically expressed Pax2 (Figs 3O and 4D), consistent with ONH expansion in *Pax2*^*GFP/GFP*^ mutants [65]. Without *Pax2*, retinal cells are unable to lock-in a neural development program expressing both RPC and ONH markers [65]. This prompted us to ask whether *Hes*^*TKO*^ and *Pax2* mutants phenocopy one another regarding the mispatterning of the ONH/OS marker *Vax1* [85–87](Fig 4E–4H). In Rax-Cre;*Hes1*^*CKO/CKO*^ and Rax-Cre;*Hes*^*TKO*^ eyes *Vax1* was shifted in the OS (arrows in Fig 4F–4G). But only in Chx10-Cre;*Hes*^*TKO*^ eyes had a *Vax1* domain that extended in the opposite direction, into the retina (Fig 4H). These data suggest that sustained *Hes1* in the ONH helps lock-in the boundary with the retina, whereas multiple *Hes* genes in adjacent RPCs are necessary for maintaining neurogenic potential.

### Notch signaling regulates both RPC growth and death

**Fig 5:**
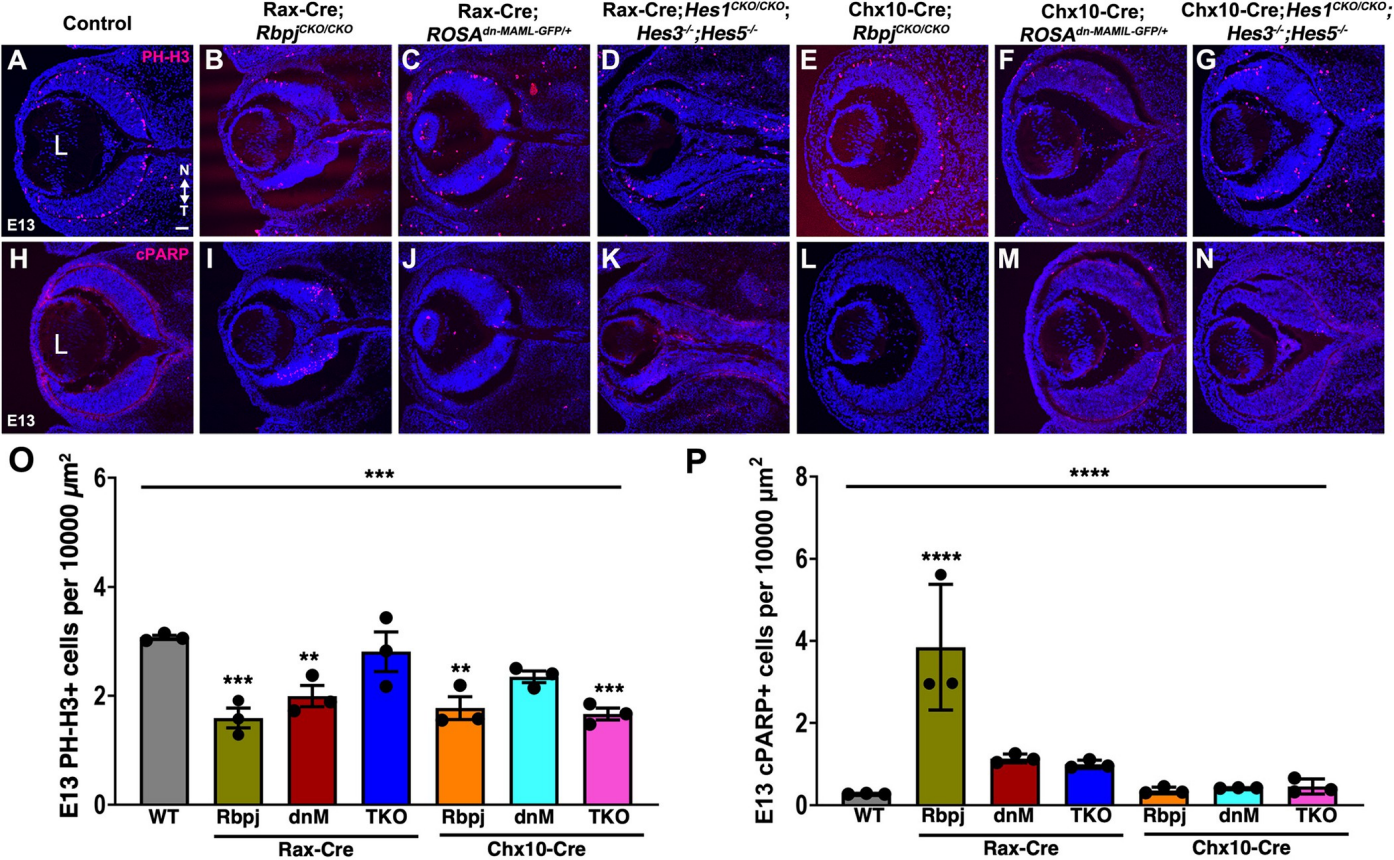
All E13.5 mutants have reduced proliferation, but only *Rbpj* mutants have excess apoptosis. (A-G) M-phase RPCs labeled with anti-PhosphoHistone-H3 (PH-H3) in red, DAPI in blue. (H-N) E13.5 cPARP+ apoptotic retinal cells in red, DAPI in blue. (O,P) Graphs display individual replicate data points normalized to optic cup area, the mean and S.E.M; Significant Welch’s ANOVA, plus pairwise comparisons to wild type (****p< 0.0001, ***p<0.001, **p<0.01). All panels are oriented nasal up (noted in A), with L = lens; scalebar in A = 50 microns; n = ≥2 sections from 3 biological replicates/genotype.

Throughout the CNS, Notch signaling stimulates progenitor cell growth and blocks neurogenesis. Reduced RPC proliferation is common to all mutants in this pathway, although the magnitude of this loss is variable (S1 Table). We expected proliferation to be reduced in the six mutants and confirmed it by quantifying PhosphoHistone H3 (PH-H3) expression within G_2_ and M-phase cells (Fig 5A–5G and 5O). Both *Rbpj* mutants have the fewest mitotic cells. There was also a modest loss of PH-H3+ cells in *Hes*^*TKO*^ mutants for the Chx10-Cre driver, but not Rax-Cre. The opposite outcome was seen in *ROSA*^*dnMAML-GFP/+*^mutants. Thus, all six mutants do not equivalently lose PH-H3+ cells, which might be due to slight differences in the degree and age of phenotypic onset between Cre mouse lines.

In the E13-E16 retina, *Notch1*, *Rbpj* and *Hes1* mutants have a significant increase in apoptosis (S1 Table) [14,16,17,50]. We used cPARP labeling to quantify dying cells among the six mutants to determine if they were equivalent (Fig 5H–5N and 5P). We observed the anticipated increase in cPARP+ cells in E13.5 Rax-Cre;*Rbpj*^*CKO/CKO*^ mutants (Fig 5I–5P), but all other genotypes were unaffected (Fig 5P). This suggests that Rax-Cre;*Hes*^*TKO*^ mutants can rescue the apoptosis phenotype previously described for *Hes1* single mutants [50]. This difference could be attributed to either *Hes1* and *Hes5* coordinated regulation of RPC target genes, or inherent interactions between retinal and ONH tissues, which impacts cell viability.

### Notch pathway regulation of prenatal retinal cell fate

**Fig 6:**
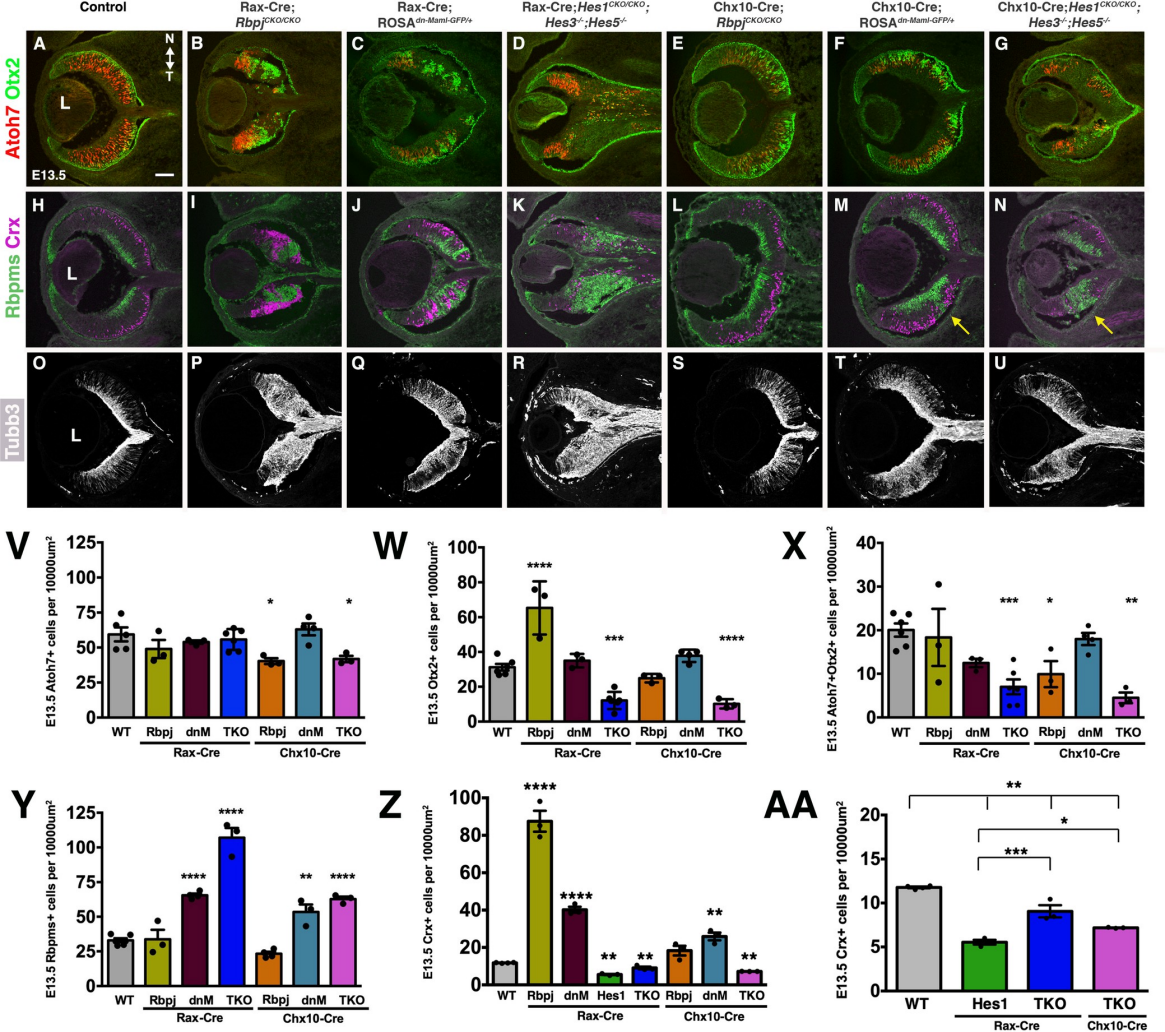
Shifts in RGC and early photoreceptor fates correlate with changes in Otx2, but not Atoh7, expression. (A-G) Atoh7-Otx2 double labeling at E13.5 highlights neurogenic defects across the allelic series and inappropriately labeled cells in D, where the retina had expanded. (H-N) E13.5 Rbpms-Crx double labeling reveals early mispatterning of RGCs (green) and photoreceptors (fuchsia). (O-U) Anti-Tubb3 labeling of E13 retinal sections emphasizes neurogenic phenotypes for all conditional mutants. All panels are oriented nasal up (noted in A), L = lens in A,H,O; scalebar in A = 50 microns. (V-Z) Quantification of Atoh7+ (V), Otx2+ (W), Atoh7+Otx2+ (X), Rbpms+ (Y), Crx+ (Z) nuclei normalized for optic cup area. Only Rax-Cre;*Rbpj* mutants have a significant increase in both Otx2+ and Crx+ cells; whereas both *Hes*^*TKO*^ mutants have a reduction of Otx2+ and Crx+ cells and an increase in Rbpms+ RGCs. (AA) Direct comparison of Crx+ cells for *Hes1* single versus *Hes*^*TKO*^ mutants (regraphed from panel Z) more clearly show a significant increase for both *Hes*^*TKO*^ mutants compared to the single mutant. Graph displays individual replicate data points normalized to optic cup area, the mean and S.E.M; Significant Welch’s ANOVA, plus individual comparisons to wild type (*p< 0.05, **p<0.01, ***p<0.001, ****p <0.0001); n = 3 biologic replicates/genotype.

The loss of Notch signaling accelerates neurogenesis among a heterogeneous population of RPCs, allowing premature differentiation of multiple fates (e.g., RGC and photoreceptor). Previous work demonstrated that deletion of *Dll1*, *Dll4*, *Notch1*, or *Rbpj* induced ectopic differentiation of both RGCs and cone photoreceptors (S1 Table) [10,13–16]. Another archetypal defect of blocking Notch signaling is the appearance of retinal rosettes full of excess Crx+ photoreceptors (S1 Table). While *Hes1* conditional mutants also contain retinal rosettes, they uniquely *downregulate* Otx2, Crx, and cone photoreceptor markers, which cannot be attributed to developmental delay [14]. This incongruity raises questions about how the Notch pathway, downstream of the ternary complex, operates in transitional RPCs relative to competence factor expression and cell fate acquisition. We also reasoned that if Hes activities are partially redundant in transitional RPCs, the simultaneous removal of multiple *Hes* repressor genes might restore or even overproduce cones. To explore these ideas, we colabeled all six mutants at E13.5, for the competence factors Atoh7 and Otx2 [24,29,32,34,88](Fig 6A–6G), and labeled adjacent sections for the RGC-marker Rbpms [89,90] and photoreceptor marker Crx [34,89–95] (Fig 6H–6N). We also qualitatively assessed ectopic neurogenesis using the general neuronal marker Tubb3 (Fig 6O–6U).

Consistent with other studies, we noted defective retinal patterning at E13.5, with rosettes containing Otx2+ or Crx+ cells residing near patches of Atoh7+ RPCs or Rbpms+ RGCs, respectively (Fig 6A–6N). Next, we quantified each nuclear marker and normalized using optic cup area (um^2^, see Methods and S5 Table) for the different mutants. Surprisingly, the proportion of Atoh7 cells was largely normal, with significantly fewer cells in only Chx10-Cre;*Rbpj*^*CKO/CKO*^ and Chx10-Cre;*Hes*^*TKO*^ eyes (Fig 6V). This did not correlate with the changes seen for either Rbpms+ RGCs or Crx+ photoreceptors (Fig 6Y). Although excess Pou4f+ RGCs were reported at E16 in a previous *Rbpj* conditional mutant study [14], here at E13.5 we found no difference in Rbpms+ RGCs, for either *Rbpj* mutant (Fig 6Y). It is plausible that ectopic RGCs in Rax-Cre;*Rbpj*^*CKO/CKO*^ eyes might rapidly die (Fig 5P), and/or there is nonautonomous rescue in Chx10-Cre;*Rbpj*^*CKO/CKO*^ eyes (S4E Fig). Alternatively, these RPCs may erroneously differentiate into neurons without fully committing to be an RGC, since there are obviously more Tubb3+ neurons in both *Rbpj* mutants, compared to control (Fig 6O–6U). Consistent with past studies of RGC genesis after blocking Notch signaling, we saw a significant increase in Rbpms+ cells for both sets of dnMAML and *Hes*^*TKO*^ mutants (Fig 6Y).

In contrast to Atoh7, the proportion of Otx2+ cells dramatically increased in Rax-Cre;*Rbpj*^*CKO/CKO*^ mutants, and significantly decreased in *Hes*^*TKO*^ mutants, with no change for dnMAML (Fig 6W). Shifts in the subset of RPCs that typically express both competence factors also reflect that the impact is on Otx2 and not Atoh7 (compare Fig 6X to Fig 6V and 6W). By contrast, there was a big increase in Crx+ cells for Rax-Cre;*Rbpj*^*CKO/CKO*^ mutants, with smaller, significant increases in most other genotypes (Fig 6Z). We also quantified Crx+ cells in Rax-Cre;*Hes1*^*CKO/CKO*^ mutants to facilitate direct comparison with both Cre-induced *Hes*^*TKO*^ mutants (Fig 6Z). This subset of data is regraphed in Fig 6AA to more easily see the partial rescue for both *Hes*^*TKO*^ mutants compared to single *Hes1* mutants. There was a simultaneous and significant increase in RGCs for all four *Hes*^*TKO*^ or dnMAML mutants (Fig 6Y). The largest increase in RGCs occurred in Rax-Cre;*Hes*^*TKO*^ eyes with expanded retinal tissue (Fig 3). Finally, it was surprising that the defects noted for Rbpms or Crx expressing cells correlate with significant changes in the cells expressing Otx2, but negatively correlate to the Atoh7+ population (Fig 6V–6X).

Direct comparison of both qualitative and quantitative defects in RGC versus Crx+ cohorts among the six mutants revealed other allele-specific defects during early retinogenesis. We found that only Rax-Cre;*Hes*^*TKO*^ mutants had displaced RGC and cone photoreceptor neurons in tissue that is normally optic stalk (Fig 6K). There were also mislocalized Rbpms+ RGCs in Chx-Cre*;ROSA*^*dnMAML-GFP/+*^ and Chx10-Cre; *Hes*^*TKO*^ eyes, akin to interkinetic nuclear migration defects reported other Notch studies (arrow Fig 6M–6N) [74]. At E16.5, we noted that only Rax-Cre;*ROSA*^*dnMAML-GFP/+*^ mutants contain more rosettes in the temporal retina (S6J Fig), suggesting a Notch-independent interaction occurred during N/T patterning that becomes more obvious over time.

It remains unclear why *Hes1* appears to promote cone genesis, rather than suppress it like other genes in the Notch pathway (Fig 6Z). One possibility is that *Hes1* regulates some aspect of cone versus rod fate choice, since postnatal *Hes1*^*-/-*^ ex vivo retinal cultures were previously described to contain premature rod photoreceptor rosettes and fewer bipolar neurons [42]. First, we verified that at E16.5 the ectopic Crx+ cells in rosettes are Thrb2+ cones (S6A–S6N Fig) and not precocious Nr2e3+ rods [96–99]. Then we tested for premature rods within the Crx+ cohort. We collected E17 littermate control and Rax-Cre;*Hes1*^*CKO/CKO*^ retinal sections and colabeled for Crx and Nr2e3, a transcription factor specifically found in nascent rods [96]. Nr2e3+ nuclei were evident within the forming outer nuclear layer (ONL) (S6O and S6P Fig) and retinal rosettes. However, the percentage of Nr2e3+Crx+ cells was identical (S6Q Fig). Therefore, the loss of cones in *Hes1* mutants cannot be attributed to accelerated rod genesis. Another explanation is that Hes1 provides temporal restriction to the Otx2 lineage to prevent prenatal bipolar neuron formation [100]. Alternatively, RGC development may accelerate in the absence of *Hes1*, depleting the availability of transitional RPCs to activate *Otx2* and adopt a cone fate.

**Fig 7:**
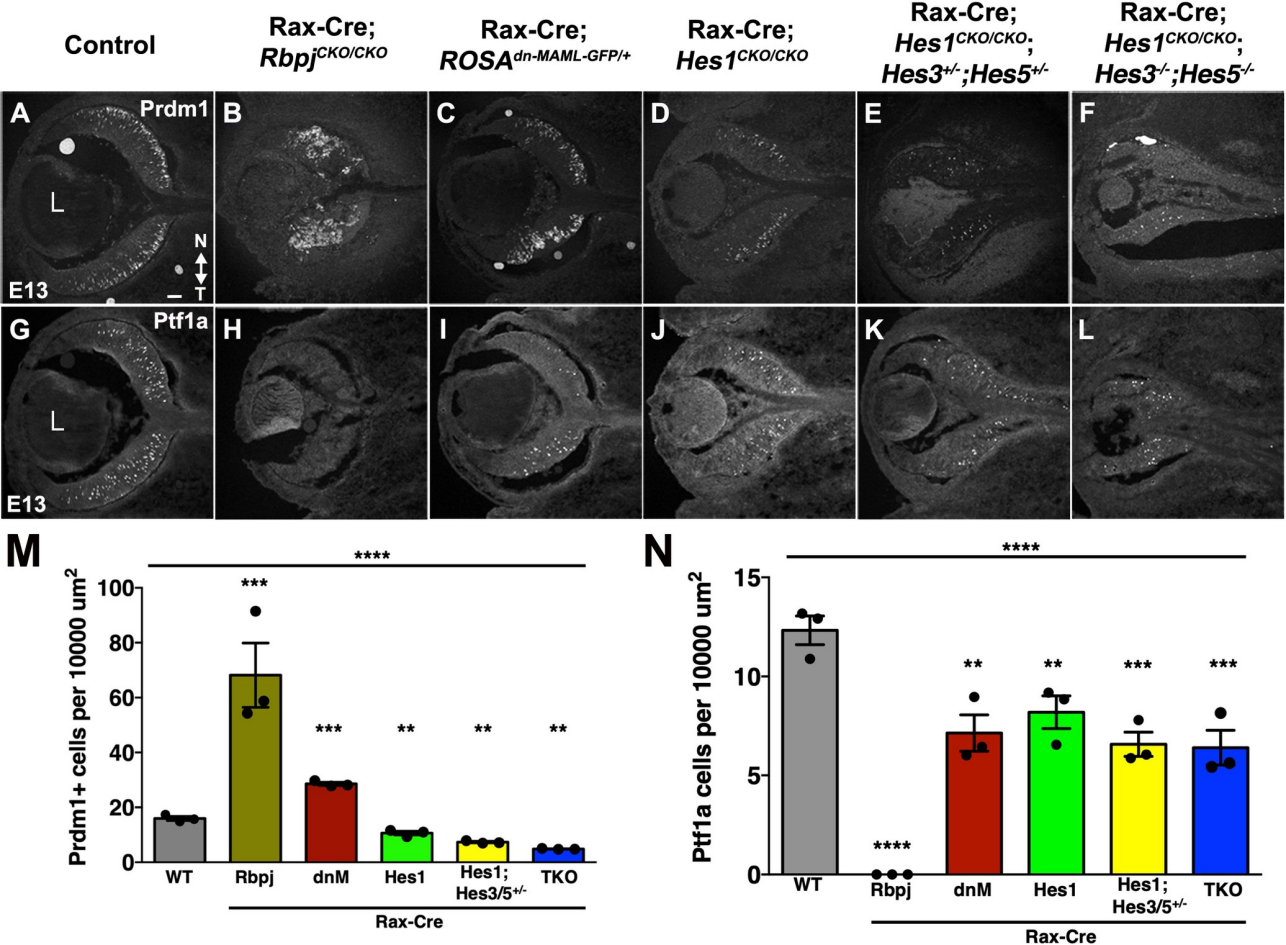
Unique role for *Rbpj* in regulation photoreceptor versus amacrine fates. Prdm1/Blimp1 (A-F) and Ptf1a (G-L) labeling of E13.5 Rax-Cre-meditated deletion of Notch pathway genes. (M,N) Strikingly, *Rbpj* mutants have both excess Prdm1+ cells and a total loss of Ptf1a-expressing cells. All other genotypes exhibit significant, but smaller, shifts in labeled populations. Graphs display individual replicate data points, the mean and S.E.M; Significant Welch’s ANOVA (****p< 0.0001; and pairwise comparisons to wild type (***p<0.001, ** p< 0.01). All panels oriented nasal up (noted in A), with L = lens in A,G; scalebar in A = 50 microns; n = ≥3 biological replicates/genotype.

Finally, we wished to understand why Rax-Cre;*Rbpj*^*CKO/CKO*^ mutants overproduce Otx2+ and Crx+ cells in such vast excess (Fig 6W–6Z). A large subset of embryonic RPCs expresses the transcription factor *Otx2* and are initially capable of producing five fates: cone, rod, amacrine, horizontal or bipolar neurons [32–34]. However, Otx2 is shut off relatively quickly in those cells that will adopt amacrine and horizontal fates. The remaining Otx2-lineage cells, which produce cones, rods and bipolar neurons [22], then activate the transcription factor Crx [91,93,94,101]. When *Otx2* activity is blocked or removed, mutant cells switch from photoreceptor/bipolar to adopt amacrine/horizontal fates [32–34]. So, we evaluated another marker directly downstream of Otx2, Prdm1/Blimp1 [102,103], that is expressed before Crx. At, E13.5 Prdm1+ cells were quantified among all Rax-Cre induced mutants, plus Rax-Cre;*Hes1*^*CKO/CKO*^ single and Rax-Cre;*Hes1*^*CKO/CKO*^;*Hes3*^*+/-*^*;Hes5*^*+/-*^ mutants for better evaluation of the relative contributions of each *Hes* gene (Fig 7A–7F and 7M). We found the greatest excess of Prdm1+ cells in *Rbpj* mutants, compared with a modest increase in Rax-Cre; *ROSA*^*dnMAML-GFP/+*^ eyes and a significant reduction in *Hes1* single or triple mutants (Fig 7M). This outcome for Prdm1 further confirms the Otx2 and Crx data, suggesting that *Rbpj* and *Hes1* act differently upstream of Otx2.

Within the early Otx2 lineage, cells transiting to amacrine or horizontal fates downregulate Otx2 as they activate the transcription factor Ptf1a [reviewed in 104]. Ptf1a is both necessary and sufficient for amacrine and horizontal fates, and when retinal cells lose this factor, they erroneously develop as RGCs and photoreceptors [105–107]. Without *Rbpj* there was a total loss of Ptf1a+ cells (Fig 7H–7N). By contrast the other mutants had only a partial loss of Ptf1a+ cells, likely reflecting a generally reduced pool of RPCs (Fig 7N). The more severe consequences of removing *Rbpj* on the amacrine pathway agree with previous studies (S1 Table), and further reinforce that Ptf1a expression depends on *Rbpj*, similar to Ptf1a target genes [17,105,107].

## Discussion

**Fig 8:**
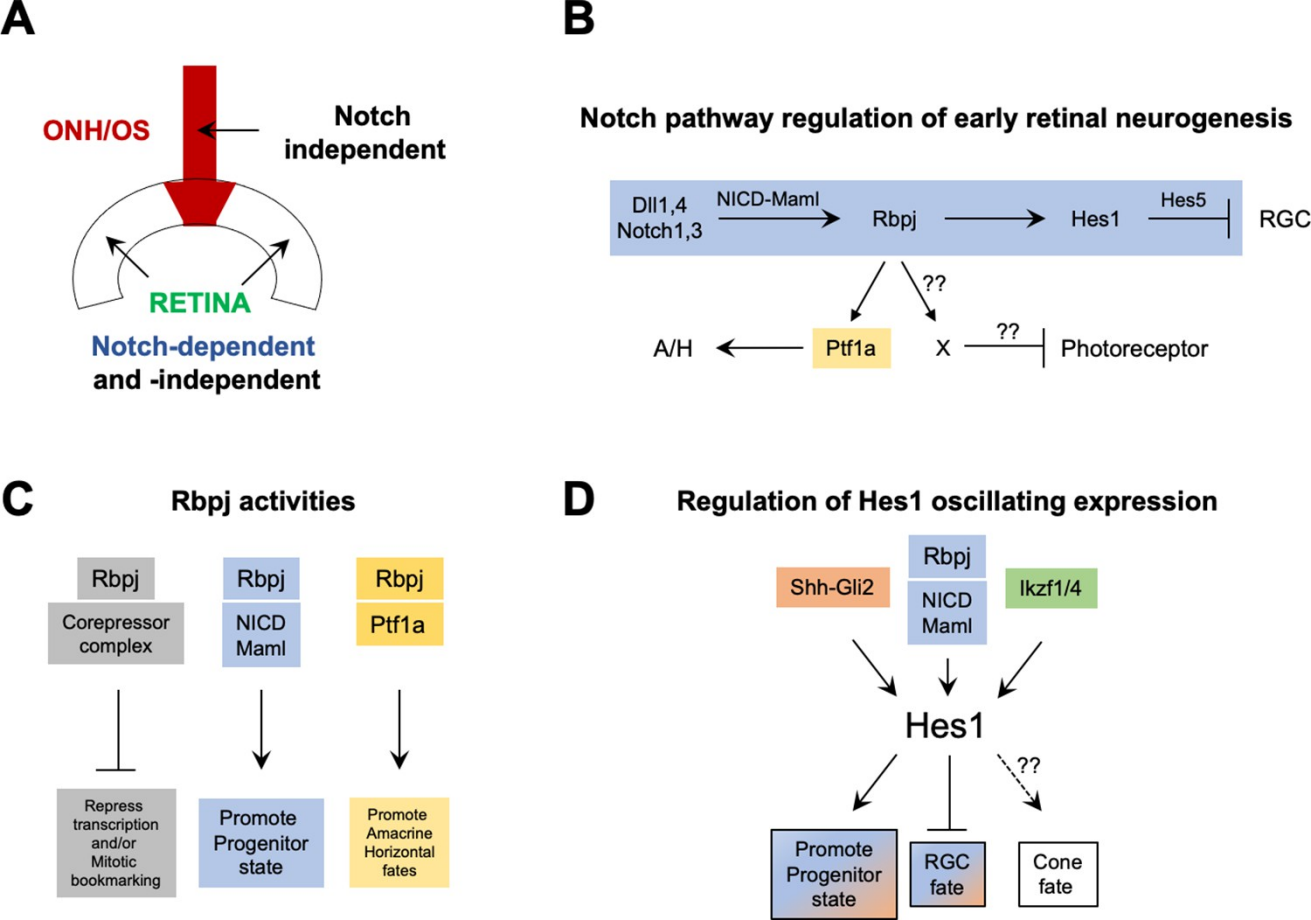
Notch pathway activities and integration points with other genetic pathways in the embryonic eye. (A) Sustained Hes1 expression in the ONH/OS does not require ternary complex gene activities (Notch-independent). RPC status and early retinal fates are Notch-dependent, but also require other inputs. (B) Canonical Notch signal (blue) blocks premature RGC differentiation, while noncanonical Rbpj activity (yellow) utilizes Notch-independent modes to also regulate photoreceptor versus amacrine/horizontal fate (A/H). (C) The Rbpj protein forms distinct protein complexes (three shown) that uniquely regulate transcription and cell fates. By sequestering Rbpj into the different complexes, the production of one cell type also impacts its availability to regulate the other early cell types. (D) Distinct Hes1 transcriptional regulators influence oscillating Hes1 expression and activity during retinal neurogenesis. Since Hes1 encodes a repressor protein, its positive effect on cones is predicted to be indirect, presumably blocking and unknown factor X that normally suppresses cone genesis.

The molecular mechanisms integrating Notch with other signaling pathways remain poorly understood. Here we directly compared the genetic requirements for ternary complex components and multiple *Hes* genes during ONH formation and the onset of retinal neurogenesis (Fig 8A). We found that only *Hes1* is required in the ONH. While all genes examined control RPC proliferation, our findings also point to particular Notch-independent activities. Although *Hes1* and *Hes5* transcriptional repressors have been compared using a variety of tools, their potential redundancy in the eye had not been tested [11,14,18,42,50,51,59,60]. *Hes1* maintains optic vesicle and cup growth, the tempo of retinogenesis, and promotes astrocyte development in ONH/OS cells. But paralogues *Hes3* and *Hes*5 have only subtle roles [18,108]. In other areas of the CNS, *Hes3* is active during oligodendrocyte maturation and interacts with STAT3-Ser and Wnt signaling pathways prior to the initiation of myelination [109,110]. Given that *Hes3* mRNA is undetectable in the embryonic retina, we propose it is relatively more important postnatally, possibly for retinal astrocyte migration, or optic nerve myelination.

### Making and keeping the retinal-glial boundary

The boundary between the retina and OS possesses many characteristics of the brain isthmus, which is comprised of slowly proliferating cells that undergo little to no neurogenesis and act as a signaling hub for adjacent neural tissues [reviewed in 39,111]. Consistent with this idea, we found *Hes1* is required for Ccnd2 expression, which is associated with prolonged cell cycles. Both the ONH and isthmus require the transcription factors *Hes1* and *Pax2*. In the eye, loss of either gene allows the retina to encroach and displace the ONH. This expansion might be due to a failure to effectively shift from fast to slow cycling kinetics or be driven by ectopic *Hes5* plus other early eye factors. In this specific context, Rax-Cre;*Hes*^*TKO*^ eyes were not much different than the loss of *Hes1* alone. Thus, sustained *Hes1* is likely sufficient for ONH formation and maintenance. We found no role for Notch regulation in ONH/OS formation since both *Rbpj* and *ROSA*^*dnMAML-GFP/+*^ animals retain a recognizable ONH with sustained Hes1 expression (Fig 8A).

Hes1 expression in the ONH/OS must be regulated by other genetic pathways. A strong candidate is the Shh pathway. Shh signaling performs an important feedback mechanism to control RGC population size. Nascent RGCs secrete Shh, which instructs RPCs to remain mitotically active via direct binding of Gli2 to activate *Hes1* transcription [52,112]. Moreover, at the optic vesicle stage of development, Shh diffuses from the ventral diencephalon midline to stimulate outgrowth of the optic cup and stalk [reviewed in 113]. Given that Wnt, Bmp and Retinoic Acid signals also regulate proximoventral optic cup and stalk outgrowth and specification [reviewed in 114], it is tantalizing to speculate that they do so by converging on Hes1 expression and/or activity. It also remains unresolved if the ONH is a signaling hub for the adjacent retina.

To delineate ONH versus retinal phenotypes, we used both Rax-Cre and Chx10-Cre drivers to test for functional redundancy of *Hes1* and *Hes5* during retinal neurogenesis. Unfortunately, the Chx10-Cre line could only produce a few robust outcomes. This was unanticipated since the Chx10-Cre driver was successfully used in past retinal analyses of *Dll1*, *Notch1*, *Hes1*, *Rbpj* and *Neurog2* function [10,13–16,50,115]. Due to mosaic expression and because fewer and fewer Cre-GFP+ cells are present as development progresses, we expect that selection pressure favored wild-type cells and their nonautonomous rescue of some of the phenotypes. Nonetheless, we uncovered distinct *Hes*^*TKO*^ phenotypes using these Cre drivers at E13.5. Only Rax-Cre;*Hes*^*TKO*^ mutants had a specific displacement of retinal tissue into the OS. The Pax6/Pax2 double positive cohort, along the retina-ONH boundary was largely missing, but the size of the mispositioned ONH was relatively normal (Fig 3). By contrast, Chx10-Cre;*Hes*^*TKO*^ mutants had a bigger Pax2 domain, further confirmed by expansion of the Vax1 ONH marker into neural retinal territory. Our interpretation is that the earlier, broader Rax-Cre mutant prevented ONH/OS cells from adopting distinct identities, thus cells remained OC-like longer, producing more retinal tissue. This is likely a Hes1-specific process. But in Chx10-Cre mutants, with Cre expression restricted to the neural side of the boundary and acting at a slightly older age, the redundant, neurogenic role of *Hes5* was revealed, since the retinal cells coexpressed neuronal and optic-stalk markers. Interestingly both phenotypes are apparent in *Pax2* mutants, suggesting that *Pax2* is upstream of Hes5, but acts parallel to *Hes1*. Future multiomic studies that characterize ONH cells, in the absence of *Hes1* or *Pax2*, will be very informative. Finally our data highlight the variable penetrance and severity of Rax-Cre versus Chx10-Cre drivers, which is instructive for future studies.

### Multiple modes regulating retinal histogenesis

Another important goal of this study was to understand how precisely *Hes1* and *Hes5* activities mirror the Notch ternary complex, which directly activates *Hes* gene transcription [reviewed in 4]. Because there are multiple ligands and Notch receptors expressed in the developing retina (Fig 8B), we focused on the requirements for *Rbpj* (Fig 8C) and to a lesser extent *Maml*. There are three *Mastermind-like* paralogues (*Maml* genes), but germline mutant analyses failed to uncover individual gene functions during embryogenesis [reviewed in 116]. Subsequently, a dominant negative isoform of MAML1 (dnMAML) was created, in which the MAML1 N-terminus forms ternary complexes with NICD and Rbpj, but cannot further interact with obligate transcriptional coactivators (e.g., p300, histone acetyltransferases) [54–57]. This has been a powerful tool in cancer biology and immunology research [54], but during retinal neurogenesis, dnMAML is less effective at blocking Notch signaling. This might be attributed to differences in expression levels relative to other studies (in vivo Cre-mediated induction here, versus plasmid or viral delivery). However, several dnMAML eye defects, namely temporal retina-specific downregulation of Hes1 and *Hes5*, Foxg1 mislocalization and an unequal appearance of photoreceptor rosettes (Figs 2, S5 and S6J) suggest that Rax-Cre;*ROSA*^*dnMAML-GFP/+*^ mutants have Notch-independent genetic interactions. In vitro proteomic studies support this idea, where dnMAML can bind to Gli and Tcf/Lef proteins [117,118]. This implies that *ROSA*^*dnMAML-GFP/+*^ retinal phenotypes may represent composite outcomes of simultaneously interfering with Notch, Shh, and/or Wnt signaling.

*Rbpj* also has Notch-independent functions (Fig 8C), the most common being its role in co-repressor protein complexes to silence transcription via DNA methylation [reviewed in 4]. Another activity is through Rbpj interactions with Ptf1a-E47 in a higher order PTF1 complex that has been studied in the pancreas, spinal cord and retina [104]. In the pancreas, PTF1 complexes can activate *Dll1*, suggesting as a feedback loop from postmitotic to mitotic cells, via Dll1 binding to Notch1 [119]. PTF1 can also directly antagonize Notch signaling in a cell autonomous and dose-dependent manner, since Ptf1a and NICD bind to the same site on the Rbpj protein [104,120]. The second scenario is likely more relevant here. It is plausible that in the retina, when a critical threshold of Rbpj protein is bound up in PTF1 complexes, it not only impacts Rbpj availability for active Notch ternary complexes, but diverts cells from photoreceptor fate choice. We conclude that *Rbpj* activity regulates early photoreceptor development in at least two ways. First, in the Notch-dependent ternary complex, *Rbpj* controls RPC division versus differentiation into neurons like photoreceptors. Second, independent of Notch, *Rbpj* prevents cells normally destined to become amacrines from erroneously developing as photoreceptors via regulation of and independent physical interaction with Ptf1a (Fig 8B and 8C).

These additional *Rbpj* and *Hes1* functions significantly complicate meaningful interpretation of our genetic data concerning Notch signaling regulation of *Otx2*. For *Rbpj* mutants, the expansion of Otx2+, Crx+, Prdm1+ cells, and cones, at the expense of Ptf1a and amacrine neurons, fits current models of mutual exclusion mentioned above [reviewed in 104]. Conversely, *Hes1* mutants produce excess RGCs and too few cones, which is essentially the opposite of *Rbpj* mutants. This might be attributed to *Hes1* loss being relatively more efficient than *Rbpj*, facilitating RPC adoption of RGC fate, which also depletes the pool available for photoreceptor formation. Alternatively, Hes1 and Rbpj may simultaneously regulate (via distinct Notch-independent activities), competence or differentiation factors, for example *Atoh7* [14,50,121]. Here we found that Atoh7 protein expression is not correlative with RGC differentiation, in agreement with, single cell transcriptomics data [24]. Instead, other competence factors, like Otx2, fluctuate as transitional RPCs adopt RGC or cone fates, with Otx2 expression becoming permanent in nascent and differentiated photoreceptors [33,34]. Does this mean that the absence of Otx2 is needed for RGC fate? We propose that the Notch genes tested here, via different modes of action, act upstream of Otx2, to influence cell cycle status while also potentially targeting other genes that enable or limit RGC formation.

When considered together, our data and other studies, point to *Hes1* as a signal integration point (Fig 8D). *Hes1* mRNA and protein are dynamic, and likely important for the establishment of cellular heterogeneity. Hes1 might convey pulsatile feedback to other oscillating molecules like *Dll1*, *Neurog2* or *Ascl1* [40,41], which could occur upstream of *Otx2*. Although circumstantial, Prdm1+ cells and rods are specifically reduced in postnatal *Neurog2* mutants, but how directly these events are linked remains to be determined [88,115]. Future studies that apply short-lived Hes reporters and single cell imaging and sequencing modalities to remaining questions about when and where Notch signaling is required will be illuminating.

## Materials and methods

### Ethics statement

All mice were housed and cared for in accordance with guidelines provided by the National Institutes of Health and the Association for Research in Vision and Ophthalmology, and conducted with approval and oversight from the UC Davis Institutional Animal Care and Use Committee (Protocols #20065 and #21839).

### Animals

Mouse strains used in this study are Hes5-GFP BAC transgenic line (*Tg(Hes5-EGFP)CV50Gsat/Mmmh* line; stock 000316-MU) [60,122]; *Hes1*^*CKO*^ allele (*Hes1*^*tm1Kag*^) maintained on a CD-1 background [53]; *Rbpj* ^*CKO/CKO*^ (*Rbpj*^*tm1Hon*^) on a *C57BL/6J* background[8]; *Pax2*^*GFP/+*^ (*Pax2*^*tm1*.*1Gdr*^) maintained on a CD-1 background [123]; ROSA26^dnMAML-GFP^ (*Gt(ROSA)26Sor*^*tm1(MAML1)Wsp*^) maintained on a C57BL/6J background [54–57]; *Hes1*^*CKO/CKO*^;*Hes3*^*-/-*^;*Hes5*^*-/-*^ (*Hes1*^*tm1Ka*^)(*Hes3*^*tm1Kag*^) (*Hes5*^*tm1Fgu*^) triple homozygous stock, maintained on CD-1 and termed "*TKO"* in this study [43,53]; *Hes3*^*-/-*^;*Hes5*^*-/-*^ mice, derived from the triple stock, with loss of *Hes3* and *Hes5* mRNA validated by whole mount in situ hybridization at E10.5; Chx10-Cre BAC transgenic line (*Tg Chx10-EGFP/cre;-ALPP)2Clc*; JAX stock number 005105) maintained on a CD-1 background [58]; and Rax-Cre BAC transgenic line (Tg(Rax-cre) NL44Gsat/Mmucd created by the GENSAT project [122], cryobanked at MMRRC UC Davis (Stock Number: 034748-UCD), and maintained on a CD-1 background. PCR genotyping was performed as described [8,43,53–58,60,122,123]. Conditional mutant breeding schemes mated one heterozygous Cre mouse to another mouse homozygous for the GeneX conditional allele to create Cre;*GeneX*^*CKO/+*^ mice. The Cre;*GeneX*^*CKO/+*^ mice were used in timed matings with *GeneX*^*CKO/CKO*^ mice (see S2 Table) and littermates lacking Cre were used as controls throughout this study. The date of a vaginal plug was assigned the age of E0.5.

### Histology and immunofluorescent labeling

P21 eyes were dissected and fixed in 4% paraformaldehyde/PBS overnight at 4°C then processed through standard dehydration steps and paraffin embedding. Four micron sections were deparaffinized using Histoclear II (National Diagnostics HS200), hydrated through graded ethanol series and either stained with Hematoxylin and Eosin (H&E), or underwent antigen unmasking in hot (95°C) 0.01M sodium citrate for 20 minutes, prior to immunofluorescent staining and imaging. For cryosection immunofluorescence, embryonic heads were fixed in 4% paraformaldehyde/PBS for 1 hour on ice, processed by stepwise sucrose/PBS incubations, and embedded in Tissue-Tek OCT. Ten micron frozen sections were labeled as in [78] with primary and secondary antibodies listed in S3 and S4 Tables. Nuclei were counterstained with DAPI.

### RNA *in situ* hybridization

DIG-labeled antisense riboprobes were synthesized from mouse *Hes5* [60], and mouse *Vax1* [85] cDNA templates. In situ probe labeling, cryosection hybridizations and color development were performed using published protocols [26,124].

### Microscopy and statistical analysis

Histologic and in situ hybridization sections were imaged with a Zeiss Axio Imager M.2 microscope, color camera and Zen software (v2.6). Antibody-labeled cryosections were imaged using a Leica DM5500 microscope, equipped with a SPEII solid state laser scanning confocal and processed using Leica LASX (v.5) plus Navigator tiling subprogram, FIJI/Image J Software (NIH) and Adobe Photoshop (CS5) software programs. All images were equivalently adjusted for brightness, contrast, and pseudo-coloring. At least 3 biologic replicates per age and genotype were analyzed for every marker, and 1–2 sections per individual were quantified via cell counting and retinal tissue area measurements (S5 Table). Sections were judged to be of equivalent depth by presence of or proximity to the optic nerve and/or characteristics of the adjacent forming lens. To normalize marker quantifications relative to tissue morphology changes, we calculated the square area (um^2^) of retinas from E13 sections, using FIJI (NIH) to trace a polygon, excluding the opening for the optic nerve [125]. The average number of marker+ cells were divided by the square micron area of the retina and graphed using Prism (GraphPadv9). For E17 retina, 11 tile scanned retinal sections for each of 3 biologic replicates/genotype were quantified, using the count tool in Adobe Photoshop CS5. Statistical analyses were performed on cells counts (S5 Table) using Prism (GraphPad v9) or Excel (v16.16.11) software, with p-values determined using one-way ANOVA and pair-wise Dunnett or pair-wise Whitney test or a Student’s T-test. p-values less than 0.05 were considered statistically significant.

## Supporting information

S1 Table Summary of Notch pathway mutant phenotypes in mouse retina. (DOCX)

S2 Table Recovery of mutant embryos/neonates at relevant stages of eye development. (DOCX)

S3 Table Validated primary antibody markers. (DOCX)

S4 Table Secondary antibody reagents. (DOCX)

S5 Table Numerical datasets underlying all graphs. (XLSX)

S1 Fig *Hes3*^*-/-*^*;Hes5*^*-/-*^ double mutants have no discernible eye phenotypes. (A,B) Number and pattern of Pou4f+ RGCs is unaltered at E13.5. (C,D) Pax6+ RPCs and mitotic Ccnd1+ cells are unaffected at E13.5. (E,F) Cdkn1b+ postmitotic RGCs and Sox9+ RPCs, RPE and ONH cells are the same between control and double mutants at E13.5. (G-J) Adult (P28) Müller glia, labeled with Sox9 (G,H) or Rlpb1/CRALBP (I,J) are also normal. All panels are vitreal down, scleral up; scalebar in A, E = 20 microns; n = 4 biologic replicates/genotype.
(TIF)

S2 Fig *Hes5* mRNA expression in E16.5 *Hes1* conditional mutants. A-C) *Hes5* inappropriately expands into the optic stalk (OS) when *Hes1* is conditionally removed with Rax-Cre (arrow in C), but not Chx10-Cre (B). L = lens; CM = ciliary margin; RPC = retinal progenitor cells; ONH = optic nerve head. Bar = 100 microns; n ≥3 per genotype.
(TIF)

S3 Fig P21 Hes triple mutants are more severe than *Hes1* single mutants. (A-D) H&E staining highlights a range of ocular defects in adult eyes. Boxed areas at higher magnification in inset. (B) Without *Hes1* an ectopic vitreal cell mass resides next to the ONH and there are sporadic retinal rosettes. (C-D) Chx10-Cre;*Hes*^*TKO*^ eyes have more extensive retinal lamination defects and abnormal ONH morphology. (E-L’) Colabeling for Tubb3 (green, neuronal processes) and Endomucin (red, endothelial cells). (I-L’) Higher magnification of boxed areas in E-H. Endomucin labeling of choroid vessels (white arrows) and blood vessels within abnormal vitreal cell masses (pink arrows). This ectopic tissue is largely devoid of Tubb3+ neurons or neural processes. Panels E,I,I’ are of an adjacent section to A; panels G,K,K’ are an adjacent section to C; panels H,L,L’ are an adjacent section to D. Asterisks in I, I’ or J,J’ indicate autofluorescent photoreceptor outer segments or red blood cells within ectopic vessels. Scalebars in A = 200 microns, E,I = 20 microns; n = 3 biologic replicates per genotype.
(TIF)

S4 Fig Relative efficiencies of Rax-Cre versus Chx10-Cre BAC Tg drivers. **(**A-A’) Normal E13.5 expression patterns for Rbpj and Hes1. (B-B") Complete loss of *Rbpj* in Rax-Cre lineage-marked cells (optic cup, RPE, ONH, OS) in red, see B’. There was also a loss of Hes1 in the cup and RPE, but not in the attenuated ONH/OS (B”). (C-D’) Anti-Rbpj and GFP labeling highlights Chx10-Cre-GFP mosaicism, with scattered GFP-negative retinal cells (red only nuclei in C, pink only in D). Chx10-Cre expression does not spread into the ONH (D). (E-F’) In Chx10-Cre;*Rbpj* mutant littermates, Rbpj+ cells are dramatically reduced, although the Hes1 retinal domain is less effected (F). Hes1 in the ONH is unaffected in Chx10-Cre animals as expected. (G-H’) At E16, Cre-GFP, Rbpj and Hes1 are normally coexpressed. (I-J’) Proportionally bigger Cre-GFP-neg regions of Chx10-Cre;*Rbpj* mutant retinas express Rbpj. In J, islands of GFP+ mutant cells are surrounded by Hes1-expressing cells, which either did not undergo Cre recombination or are wild type cells that eventually outcompete and subsequently outnumber the mutant cells. Scalebar in A, C = 50 microns, L = lens in A,B; n = 3 biologic replicates/genotype.
(TIF)

S5 Fig Nasal-temporal patterning is normal in *Notch* pathway mutants. (A-G) At E13.5 Foxg1, in the nasal retina, is properly patterned among nearly all mutants. Only Rax-Cre;*Hes*^*TKO*^ eyes (D), showed Foxg1 expansion into the optic stalk, consistent with other RPC markers (Figs 1G,3D,3K), where it remained biased to the nasal portion of the retina and optic stalk. (H-M) At E16.5, all mutants have nasally-restricted Foxg1 expression, except Rax-Cre;*ROSA*^*dnMAMl1-GFP/+*^ retinas that have some Foxg1+ nuclei present on the temporal side and within the adjacent subretinal space (arrow in J). All panels oriented nasal up (noted in A) and brain to the right; with L = lens in A,H; scalebar in A, H = 50 microns; n = 3 biologic replicates/genotype.
(TIF)

S6 Fig E16.5 Notch pathway mutant cone photoreceptor rosettes. (A-G) Immunostaining for the cone-specific Thrb2 marker at E16.5. (H-N) Crx-Rbpms colabeling of adjacent E16.5 sections highlights the abundance of RGCs and cones relative to other unlabeled cells, as well as photoreceptor rosettes surrounded by RGCs. Panels A-N oriented nasal up, n = ≥3 biological replicates/genotype. (O,P) Crx-Nr2e3 double labeling of E17.5 control and Rax-Cre;*Hes1*^*CKO/CKO*^ retinas. (Q) Quantification of colabeled cells within the Crx population indicates no difference in nascent Nr2e3+ rods between genotypes. Panels A-N oriented nasal up (indicated in A), panels O,P oriented scleral up; graphical data in Q represents 13 control and 11 tile scanned composite images from 3 biologic replicates/genotype, displaying individual replicate data points, mean and standard deviation. A student t-test, with unequal variance was used to calculate a p-value in Q. Scalebars in A = 50 microns, P = 20 microns, L = lens in A,H; ONL = outer nuclear layer.
(TIF)
