# Supplementary material for: Notch pathway mutants do not equivalently perturb mouse embryonic retinal development
Source: PLoS Genet. 2023 Sep 26;19(9):e1010928. doi: 10.1371/journal.pgen.1010928 (PMC10522021; doi:10.1371/journal.pgen.1010928)
Supplement: S1 Table — (DOCX) [file pgen.1010928.s001.docx]

**S1 Table: Summary of mouse Notch signaling pathway retinal phenotypes**

| **Mouse Gene** | **Mutation** | **Progenitors** | **Apoptosis** | **Retinal**  **Ganglion Cells**  **Neurons** | **Photoreceptors** | **Other Postnatal**  **Cell types** | **Morphologic**  **defects** | **References** |
| --- | --- | --- | --- | --- | --- | --- | --- | --- |
| **Ligands^1^** | | | | | | | | |
| *Dll1* | Conditional  α Cre | **↓** | NT | **↑ RGCs** and Neurons | **Cones** Normal  **Rods** NT | Normal | R | 1 |
| *Dll1* | Conditional  Chx10 Cre | **↓** | **↑** | **↑ RGCs** and Neurons | NT | NT | R  OLM | 2 |
| *Dll4* | Conditional  Six3 Cre FoxN4 Cre | **↓** | **↑** ^E14^ | **RGCs** NT  **↑** Neurons | **↑ Cones** ^E14.5^  **↑ Rods** ^>P0^ | **↓ AC** **↓ BP**  **↓ MG** | Thinner retina  R | 3 |
| **Receptors^1^** | | | | | | | | |
| *Notch1* | Germline  Lethal ≥E10 | NA | NA | NA | NA | NA | NA | 4 |
| *Notch1* | Conditional  α Cre | **↓** | **↑** ^E14^ | **↓ RGCs** and Neurons | **↑ Cones** ^E13.5^  **↑ Rods** ^≥ P0^ | NT | M, R | 5 |
| *Notch1* | Conditional  Chx10 Cre | **↓** | NT | **↓** **RGCs** and  Neurons | **↑ Cones** ^≥E13.5^ | NT | M, R | 6 |
| *Notch1* | Conditional  ≥ P0 viral Cre | NA | NT | NA | **↑ Rods** | **↓ BP**  **↓ MG** | R | 6,7 |
| *Notch3* | Germline | Normal | NT | Normal | Normal | NT | NT | 8 |
| *Notch1*  *Notch3* | Conditional (α)  Germline | **↓** | NT | **↑ RGCs** and Neurons | **↑ Cones** ^E13.5^ | NT | NT | 8 |

| **Ternary complex** | | | | | | | | |
| --- | --- | --- | --- | --- | --- | --- | --- | --- |
| *Rbpj* | Germline  Lethal ≥E8-9 | NA | NA | NA | NA | NA | NA | 4 |
| *Rbpj* | Conditional  α Cre | **↓** | **↑** ^E16^ | ↑ **RGCs** and Neurons | **↑ Cones** ^E13.5^  **↑ Rods** ^≥ P0^ | **↓ BP**  **↓ MG** | M, R | 9 |
| *Rbpj* | Conditional  Chx10 Cre | **↓** | **↑** ^E14^ | ↑ **RGCs** and Neurons | **↑ Cones** ^≥ P0^  **↑ Rods** ^≥P0^ | **↓ AC ↓ BP**  **↓ MG** | M, R  Laminar defect | 10 |
| *Maml1* | Germline | NT | NT | NT | NT | NT | Normal | 11,12 |
| *Maml3* | Germline | NT | NT | NT | NT | NT | Normal | 13 |
| *Maml1*  *Maml3* | dbl Germline  Lethal ≥E10 | NA | NA | NA | NA | NA | NA | 13 |
| **Effectors** | | | | | | | | |
| *Hes1* | Germline  Lethal ≥E15 | **↓** | NT | **RGCs**  NT  **↑** Neurons | **Cones** NT  Explants **↑ Rods** | Explants  **↓ BP** **↓ MG** | M, R  ONH | 14 |
| *Hes1* | Germline  Lethal ≥E15 | **↓** | NT | **↑ RGCs** and Neurons | NT | Explants  **↓ MG** | M, R  OLM; ONH | 15 |
| *Hes1* | Germline  Lethal ≥E15 | **↓** | NT | **↑ RGCs** and Neurons | NT | NT | M | 16 |
| *Hes1* | Conditional  Rax Cre  Chx10 Cre | **↓** | **↑** ^E16^ | **↑ RGCs** and Neurons | **↓ Cones** ^E13.5^  Rods NT | **↑ BP**  **↓ MG** | M, R  OLM  ONH | 17 |
| *Hes3* | Germline | NT | NT | NT | NT | NT | Normal | 18 |
| *Hes5* | Germline | Normal | Normal | NT | NT | **↓ MG** | Normal | 19 |

^1^ *Jag1* and *Notch2* genes are required for lens fiber cell differentiation and ciliary body/iris formation; *Notch2* is required in the RPE

Abbreviations: NT = not tested; NA = not applicable; M = microphthalmia; R = retinal rosettes; RGC = retinal ganglion cells; AC = amacrines;

BP = bipolars; MG = Muller glia; OLM = discontinuities in Outer Limiting Membrane; ONH = Expansion of retina into Optic Nerve Head/optic stalk

**References**

1. Riesenberg AN, Brown NL. Cell autonomous and nonautonomous requirements for Delltalike1 during early mouse retinal neurogenesis. Dev Dyn. 2016;245(6):631-40.

2. Rocha SF, Lopes SS, Gossler A, Henrique D. Dll1 and Dll4 function sequentially in the retina and pV2 domain of the spinal cord to regulate neurogenesis and create cell diversity. Dev Biol. 2009;328(1):54-65.

3. Luo H, Jin K, Xie Z, Qiu F, Li S, Zou M, Cai L, Hozumi K, Shima DT, Xiang M. Forkhead box N4 (Foxn4) activates Dll4-Notch signaling to suppress photoreceptor cell fates of early retinal progenitors. Proc Natl Acad Sci U S A. 2012;109(9):E553-62.

4. de la Pompa JL, Wakeham A, Correia KM, Samper E, Brown S, Aguilera RJ, Kakano T, Honjo T, Mak TW, Rossant J, Conlon RA. Conservation of the Notch signaling pathway in mammalian neurogenesis. Development. 1997;124:1139-48.

5. Yaron O, Farhy C, Marquardt T, Applebury M, Ashery-Padan R. Notch1 functions to suppress cone-photoreceptor fate specification in the developing mouse retina. Development. 2006;133(7):1367-78.

6. Jadhav AP, Mason HA, Cepko CL. Notch 1 inhibits photoreceptor production in the developing mammalian retina. Development. 2006;133(5):913-23.

7. Mizeracka K, DeMaso CR, Cepko CL. Notch1 is required in newly postmitotic cells to inhibit the rod photoreceptor fate. Development. 2013;140(15):3188-97.

8. Maurer KA, Riesenberg AN, Brown NL. Notch signaling differentially regulates Atoh7 and Neurog2 in the distal mouse retina. Development. 2014

9. Riesenberg AN, Liu Z, Kopan R, Brown NL. Rbpj cell autonomous regulation of retinal ganglion cell and cone photoreceptor fates in the mouse retina. J Neurosci. 2009;29(41):12865-77

10. Zheng MH, Shi M, Pei Z, Gao F, Han H, Ding YQ. The transcription factor RBP-J is essential for retinal cell differentiation and lamination. Molecular brain. 2009;2:38

11. Mastermind-1 is required for Notch signal-dependent steps in lymphocyte development in vivo. Oyama T, Harigaya K, Muradil A, Hozumi K, Habu S, Oguro H, Iwama A, Matsuno K, Sakamoto R, Sato M, Yoshida N, Kitagawa M. Proc Natl Acad Sci U S A. 2007 Jun 5;104(23):9764-9

12. The transcriptional coactivator Maml1 is required for Notch2-mediated marginal zone B-cell development. Wu L, Maillard I, Nakamura M, Pear WS, Griffin JD. Blood. 2007 Nov 15;110(10):3618-23. \

13. Mastermind-like 1 (MamL1) and mastermind-like 3 (MamL3) are essential for Notch signaling in vivo. Oyama T, Harigaya K, Sasaki N, Okamura Y, Kokubo H, Saga Y, Hozumi K, Suganami A, Tamura Y, Nagase T, Koga H, Nishimura M, Sakamoto R, Sato M, Yoshida N, Kitagawa M. Development. 2011 Dec;138(23):5235-46.

14. Tomita K, Ishibashi M, Nakahara K, Ang S-L, Nakanishi S, Guillemot F, et al. Mammalian hairy and Enhancer of split homolog 1 regulates differentiation of retinal neurons and is essential for eye morphogenesis. Neuron. 1996;16:723-34

15. Takatsuka K, Hatakeyama J, Bessho Y, Kageyama R. Roles of the bHLH gene Hes1 in retinal morphogenesis. Brain Res. 2004;1004(1-2):148-55.

16. Lee HY, Wroblewski E, Philips GT, Stair CN, Conley K, Reedy M, et al. Multiple requirements for Hes 1 during early eye formation. Dev Biol. 2005;284(2):464-78.

17. Bosze B, Moon MS, Kageyama R, Brown NL. Simultaneous Requirements for Hes1 in Retinal Neurogenesis and Optic Cup-Stalk Boundary Maintenance. J Neurosci. 2020;40(7):1501-13.

18. Hirata H, Tomita K, Bessho Y, Kageyama R. Hes1 and Hes3 regulate maintenance of the isthmic organizer and development of the mid/hindbrain. EMBO J. 2001;20(16):4454-66.

19. Hojo M, Ohtsuka T, Hashimoto N, Gradwohl G, Guillemot F, Kageyama R. Glial cell fate specification modulated by the bHLH gene Hes5 in mouse retina. Development. 2000;127(12):2515-22.
