# Supplementary material for: Notch pathway mutants do not equivalently perturb mouse embryonic retinal development
Source: PLoS Genet. 2023 Sep 26;19(9):e1010928. doi: 10.1371/journal.pgen.1010928 (PMC10522021; doi:10.1371/journal.pgen.1010928)
Supplement: S2 Table — (DOCX) [file pgen.1010928.s002.docx]

**S2 Table: Mendelian inheritance of evaluated Notch pathway mutant alleles.**

| **Mouse mating** | **Expected**  **Mutant Ratio** | **E10.5-E11** | **E13.5** | **E16.5** | **P0** | **P21** |
| --- | --- | --- | --- | --- | --- | --- |
|  |  |  |  |  |  |  |
| Chx10Cre;*Rbpj^CKO/+^*  **X**  *Rbpj^CKO/CKO^* | 25% | 22%  14 / 65 from  6 litters | 36%  14 / 39 from  4 litters | 22%  11 / 51 from  5 litters | 23%  3 / 13 from  1 litter | 20%  3 / 15 from  6 litters |
|  |  |  |  |  |  |  |
| Rax-Cre;*Rbpj^CKO/+^*  **X**  *Rbpj^CKO/CKO^* | 25% | 24%  8 / 34 from  10 litters | 9%  3 / 33 from  6 litters | 31%  5 / 16 from  5 litters | 28%  1 / 6 from  2 litters | 30%  3 / 10 from  2 litters |
|  |  |  |  |  |  |  |
| Chx10Cre;*Hes* triple het ***^a^***  **X** *Hes* triple ***^b^*** | 12.5%***^c^*** | No  data | 21%  19 / 89 from  12 litters | 8%  4 / 52 from  11 litters | 14%  6 / 42 from  4 litters | 16%  14 / 96 from  6 litters |
|  |  |  |  |  |  |  |
| Rax Cre;*Hes* triple het ***^a^***  **X**  *Hes* triple ***^b^*** | 12.5%***^c^*** | 8%  6 / 75 from  14 litters | 14%  9 / 66 from  10 litters | Not viable  0 / 10 from  3 litters | No  data | No  data |
|  |  |  |  |  |  |  |
| Chx10Cre  **X**  *ROSA^dnMaml1-GFP/ dnMaml1-GFP^* | 50% | 55%  2 / 36 from  5 litters | 54%  13 / 24 from  3 litters | 66%  10 /15 from  2 litters | 50%  3/6 from  1 litter | 50%  4/8 from  1 litter |
|  |  |  |  |  |  |  |
| Rax-Cre  **X**  *ROSA^dnMaml1-GFP/ dnMaml1-GFP^* | 50% | 58%  21 / 36 from  6 litters | 52%  14 / 27 from  6 litters | 55%  11 / 20 from  3 litters | 44%  12 / 27 from  5 litters | 43%  3 / 7 from  1 litter |

^a^ *Hes* triple het = *Hes1^CKO/+^;Hes3^KO/+^;Hes5^KO/+^*

^b^ *Hes* triple (Hes^TKO)^ = *Hes1^CKO/CKO^;Hes3^KO/KO^;Hes5^KOKO^*

^c^ *Hes3* and *Hes5* genes <1Mb apart. Mutant alleles segregate together with no recombinants found by PCR genotyping (n = 52 litters)
