# Supplementary material for: Notch pathway mutants do not equivalently perturb mouse embryonic retinal development
Source: PLoS Genet. 2023 Sep 26;19(9):e1010928. doi: 10.1371/journal.pgen.1010928 (PMC10522021; doi:10.1371/journal.pgen.1010928)
Supplement: S3 Table — (DOCX) [file pgen.1010928.s003.docx]

**S3 Table: Validated Primary antibodies.**

Authentication methods used: 1: Antibody identification at http://antibodyregistry.org; 2: Genetic validation method; 3: Comparison with known published patterns in the literature; 4: Co-labeling of tagged protein with endogenous protein.

| Antigen | Source | Host | Catalog #  (clone) | Dilution Used | Application | Authentication | Antibody ID | References (PMID) |
| --- | --- | --- | --- | --- | --- | --- | --- | --- |
| Ap2alpha  Tfap2a | DSHB | Mouse clone 3B5 | 3B5 | 1:50  conc sup | IF | 1,2,3 | AB_528084 | >10 |
| Atoh7 | Novus | Rabbit | NBP1-88639  Lot R12065 | 1:500 | IF | 1 2 3 4 | AB_11034390 | 29225067 |
| RFP-Biotin | Abcam | Rabbit | AB34771 | 1:1000 | IF | 1 3 | AB_777699 | 26587807  28641110  28817801 |
| Crx | Sigma/Prestige | Rabbit | HPA036762  Lot # R34426 | 1:1000 | IF | 1 2 3 4 | AB_10673663 | 31949107 |
| Cdkn1b* (p27) | BD/Invitrogen | Mouse | clone 57 | 1:100 | IF | 1 2 3 | AB_1075241 | 16690048 |
| Cyclin D1  (Ccnd1) | Neomarkers /  Lab Vision | Rabbit | RM-9104-S1  Clone: SP4 | 1:100 | IF | 1 3 | AB_149913 | 15166673 |
| Cyclin D2  (Ccnd2) | Santa Cruz | Rat | SC-452  Clone 34B1-3 | 1:200 | IF | 1 2 3 | AB_627350 | 27957530  29533784 |
| Arr3  cone Arrestin | EMD Millipore | Rabbit | AB15282 | 1:700 | IF | 1 3 | AB_11210270 | 26453550  25798616  24651551 |
| cPARP  (Mus-specific) | Cell Signaling | Rabbit | 9544 | 1:500 | IF | 1 3 | AB_2160724 | 27353360  30581080  31000436 |
| Endomucin | Santa Cruz | Rat | sc-65495 | 1:50 | IF | 1 3 | AB_2100037 | >10 |
| Foxg1 | Abcam | Rabbit | AB196868 | 1:1000 | IF | 1 3 | AB_2892604 | 32024767 |
| GFP | Aves | Chicken | GFP-1020 | 1:3000 | IF | 1 3 | AB100000240 | >100 in  Registry |
| Glutamine Synthetase | EMD Millipore | Mouse | MAB302  (Clone GS-6) | 1:1000 | IF | 1 3 | AB_309678 | 26283925  26527153  25907681 |
| Hes1 | Cell Signaling | Rabbit | 11988  D6P2U | 1:500 | IF | 1 2 3 4 | AB_2728766 | 29681454  30059908 |
| Hes1 | Brown lab | Rabbit | N/A | 1:500 | IF | 2 3 4 | No data | 30059908  28675662  25100656  19828801 |
| Jagged1* (C20) | Santa Cruz | Goat | SC-6011 | 1:500 | IF | 1 2 3 4 | AB_649689 | 19389370  30890522 |
| Lhx2 | GENETEX | Rabbit | GTX129241 | 1:500 | IF | 1 3 | AB_2783558 | 31135891 |
| Mitf | ThermoFisher | Mouse IgG1 | MS-771-P1 | 1:100 | IF | 1 3 | AB_141542 | 31949107  33428890 |
| Nr2e3 | R&D Systems | Mouse IgG2a | PP-H7223-00  (Clone #H7223) | 1:200 | IF | 1 3 | AB_1964331 | 29233477  32023475 |
| Opn1mw | Cheryl Craft | Rabbit | N/A | 1:1000 | IF | 1 3 | No data | 12853434 |
| Opn1sw | Cheryl Craft | Rabbit | N/A | 1:1000 | IF | 1 3 | No data | 12853434 |
| Otx2-Biotin | R&D Systems | Goat | BAF1979 | 1:1000 | IF | 1 3 | AB_2157171 | 28089909  30048641 |
| Pou4f/  Brn3 * | Santa Cruz | Goat | SC 6026 | 1:50 | IF | 1 3 | AB_673441 | 18626943  21246546  21452196  + 12 more |
| Pax2 | Biolegend  Covance | Rabbit | 901001  PRB-276P | 1:1000 | IF | 1 2 3 | AB_2565001  AB_291611 | 2043750  2073760  2247382  3065975 |
|  |  |  |  |  |  |  |  |  |
| Pax6 | Biolegend  Covance | Rabbit | 901301  PRB-278P | 1:1000 | IF | 1 2 3 4 | AB_2565003  AB_291612 | 28132835  + 24 more  >40 in Registry |
| Pax6 | Santa Cruz | Mouse (IgG_1)_ | SC-32766  (clone AD2.38) | 1:50 | IF | 1 2 3 | AB_628107 | 25100656 |
| Phospho Histone H3 | EMD Millipore | Rabbit | 06-570  Lot# DAM 1416518 | 1:500 | IF | 1 3 | AB_310177 | 17447250 18205207 18273885  >70 in registry |
| Prdm1/  Blimp1 | Santa Cruz | Rat | sc-47732  (clone 6D3) | 1:100 | IF | 1,2,3 | AB_628168 | 30184502  39463012  30850343 |
| Prox1 | Chemicon/Millipore | Rabbit | AB 5475 | 1:2000 | IF | 1,2,3 | AB_177485 | 17183554  +10 others |
| Ptf1a | Chris Wright | Rabbit | N/A | 1:2000 | IF | 1,2,3 | No data | 17075007 |
| Rax | TaKaRa | Rabbit | M228 | 1:1000 | IF | 3 | No data | 2911759  31949107 |
| Rbpj | Cosmo Bio Japan | Rat | SIM-2ZRBP1  Clone T6709 | 1:100 | IF | 1 2 3 | No data | 22275127  29977079  30059908 |
| Rbpms | PhosphoSolutions  EMD Millipore | Guinea Pig | 1832-RPBMS  ABN1376 | 1:500 | IF | 1 3 | AB_2492226  AB_2687403 | 25631988 27391320  +10 others |
| Rho | EMD Millipore | Mouse  clone 1D4 | MAB5356 | 1:1000 | IF | 1 3 | AB_11215453 | 30401922 |
| Rlbp1  CRALBP | ThermoFisher | Mouse  clone B2 | MA1-813 | 1:100 | IF | 1 3 | AB_2178528 | 11839540  11909957  +18 others |
| Rxrg* | Santa Cruz | Rabbit | SC-774 | 1:200 | IF | 1 3 | AB_2270041 | 39332629  30943581 |
| Six3 | Rockland | Rabbit | 600-401-A26 | 1:500 | IF | 1 2 3 4 | AB_11180063 | 30485816 |
| Sox2 | Millipore | Rabbit | AB5603  Lot # LV1395178 | 1:400 | IF | 1 2 3 | AB_2286686 | 16680766  >40 in registry |
| Sox9 | Millipore | Rabbit | AB5535 | 1:200 | IF | 1 3 | AB2239761 | 18626943  + 33 more |
| Thrb2 | Douglas Forrest NIH | Rabbit | N/A | 1:3000 | IF | 2 3 | No data | 19282790 |
| Tubb3 | Biolegend  Covance | Rabbit | 802001  PRB-435P | 1:1000 | IF | 1 3 | AB_2564645  AB_291637 | 29247817  16874803  + others |
| Tubb3 | Biolegend  Covance | Mouse  (IgG_2A_) | 801213  MMS-435P | 1:1000 | IF | 1 3 | AB_2564645  AB_291637 | 30695697  31209173  + others |
| Vsx2/Chx10 | Exalpha | Sheep | X1180P  N-terminus | 1:500 | IF | 1 3 | AB_2314191 | 19827163  27565351 |
| Vsx2/Chx10 | Sigma/Prestige | Rabbit | HPA003436 | 1:500 | IF | 1 3 | AB_1078523 | none |
| ZO1 | Millipore | Rat | MABT11  Clone R40.76 | 1:500 | IF | 1 3 | AB_10616098 | 25078648,  23897660,  23991284 |
|  |  |  |  |  |  |  |  |  |

*: Discontinued; IF = Immunofluorescence; N/A = not applicable
