# Supplementary material for: Notch pathway mutants do not equivalently perturb mouse embryonic retinal development
Source: PLoS Genet. 2023 Sep 26;19(9):e1010928. doi: 10.1371/journal.pgen.1010928 (PMC10522021; doi:10.1371/journal.pgen.1010928)
Supplement: S4 Table — (DOCX) [file pgen.1010928.s004.docx]

**S4 Table. Secondary or Tertiary antibodies used in this study.**

| Antibody | Source | Host | Catalog Number | Dilution Used |
| --- | --- | --- | --- | --- |
| Anti Chicken IgY  Alexa 488 | ThermoFisher | Goat | A-11039 | 1:1000 |
| Anti-Sheep IgG  Alexa 594 | Jackson Immuno. | Donkey | 713-585-147 | 1:500 |
| Anti-Guinea Pig IgG (H+L) Alexa 647 | ThermoFisher | Goat | A-21450 | 1:200 or 1:500 |
| Anti-Goat IgG  Alexa 594 | ThermoFisher | Donkey | A11058 | 1:500 |
| Anti-Goat IgG (H+L)  Alexa 647 | Jackson Immuno. | Donkey | 705-605-147 | 1:500 |
| Anti-Rabbit IgG  Alexa 594 | ThermoFisher | Goat | A11037 | 1:500 |
| Anti-Rabbit IgG (H+L)  Cy3 | Jackson Immuno. | Goat | 111-165-144 | 1:500 |
| Anti-Rabbit IgG  Biotin-SP | Jackson Immuno. | Donkey | 711-005-152 | 1:1000 |
| Anti-Mouse IgG (H+L)  Alexa 647 | ThermoFisher | Goat | A21236 | 1:200 |
| Anti-Mouse IgG_1_  Alexa 647 | ThermoFisher | Goat | A212540 | 1:200 |
| Anti-Mouse IgG2a  Alexa 647 | Jackson Immuno | Goat | 115-605-206 | 1:500 or 1:1000 |
| Anti-Rat IgG  Alexa 594 | Jackson Immuno. | Donkey | 712-586-153 | 1:500 |
| Anti-Rat IgG (H+L)  Alexa 647 | Jackson Immuno. | Donkey | 712-605-153 | 1:200 |
| Streptavidin Alexa 594 | Jackson Immuno. | Not applicable | S32356 | 1:1000 |
| Streptadivin Alexa 647 | Molecular Probes | Not aplicable | S32357 | 1:500 |
| DAPI 1 mg/ml | Sigma | Not applicable | D9542 | 1:500 |
