# Supplementary figures and images for: Notch pathway mutants do not equivalently perturb mouse embryonic retinal development
Source: PLoS Genet. 2023 Sep 26;19(9):e1010928. doi: 10.1371/journal.pgen.1010928 (PMC10522021; doi:10.1371/journal.pgen.1010928)

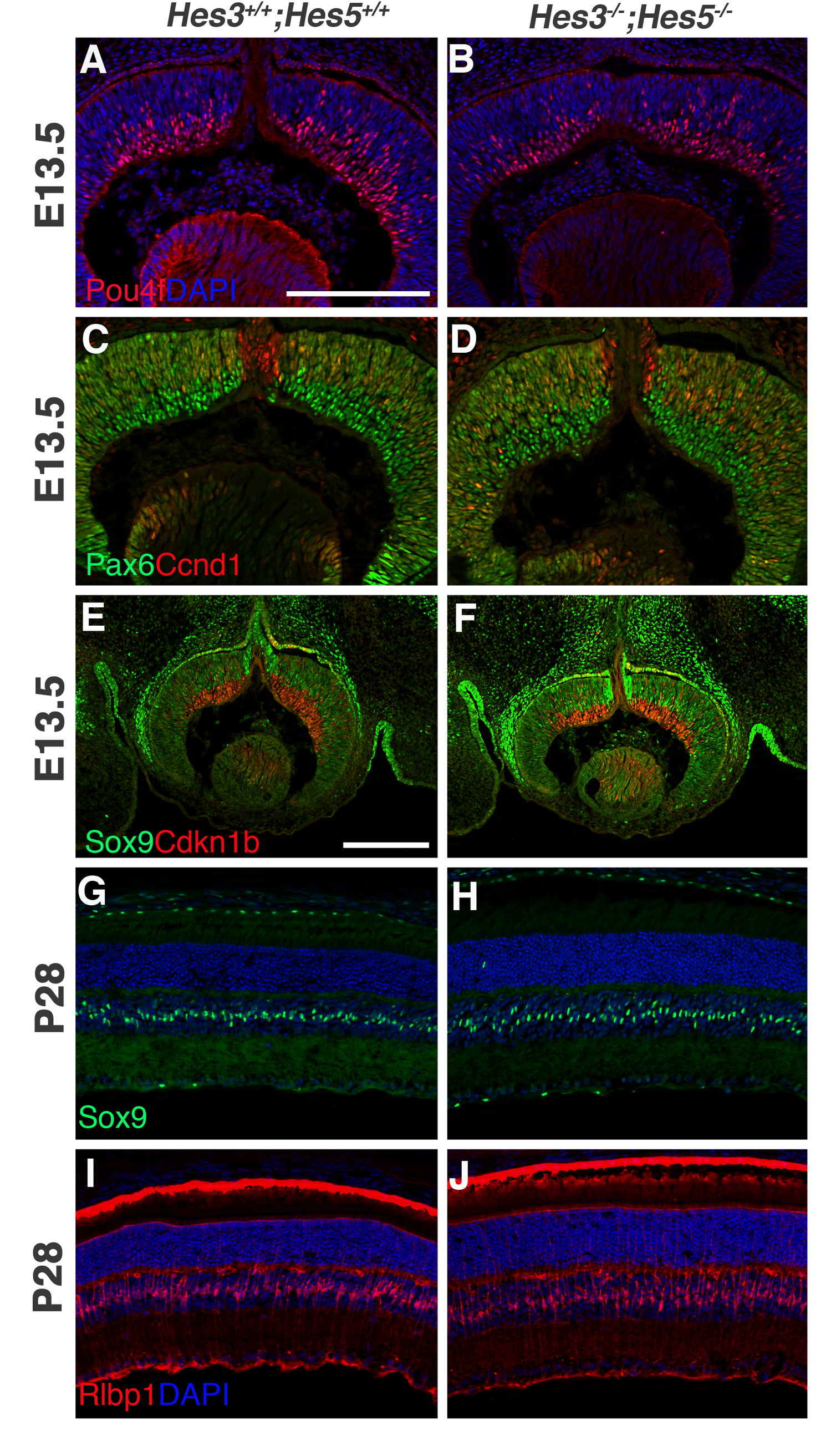

Supplement: S1 Fig — (A,B) Number and pattern of Pou4f+ RGCs is unaltered at E13.5. (C,D) Pax6+ RPCs and mitotic Ccnd1+ cells are unaffected at E13.5. (E,F) Cdkn1b+ postmitotic RGCs and Sox9+ RPCs, RPE and ONH cells are the same between control and double mutants at E13.5. (G-J) Adult (P28) Müller glia, labeled with Sox9 (G,H) or Rlpb1/CRALBP (I,J) are also normal. All panels are vitreal down, scleral up; scalebar in A, E = 20 microns; n = 4 biologic replicates/genotype. (TIF) [file pgen.1010928.s006.tif]

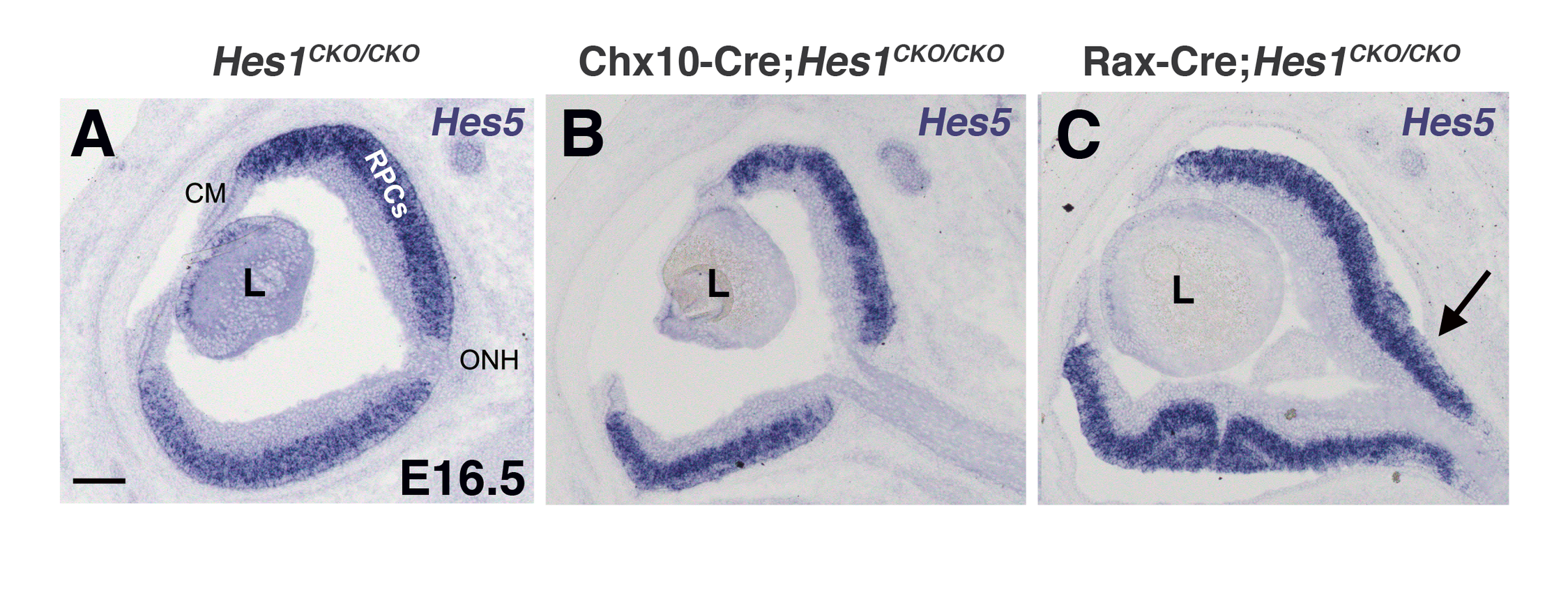

Supplement: S2 Fig — A-C) Hes5 inappropriately expands into the optic stalk (OS) when Hes1 is conditionally removed with Rax-Cre (arrow in C), but not Chx10-Cre (B). L = lens; CM = ciliary margin; RPC = retinal progenitor cells; ONH = optic nerve head. Bar = 100 microns; n ≥3 per genotype. (TIF) [file pgen.1010928.s007.tif]

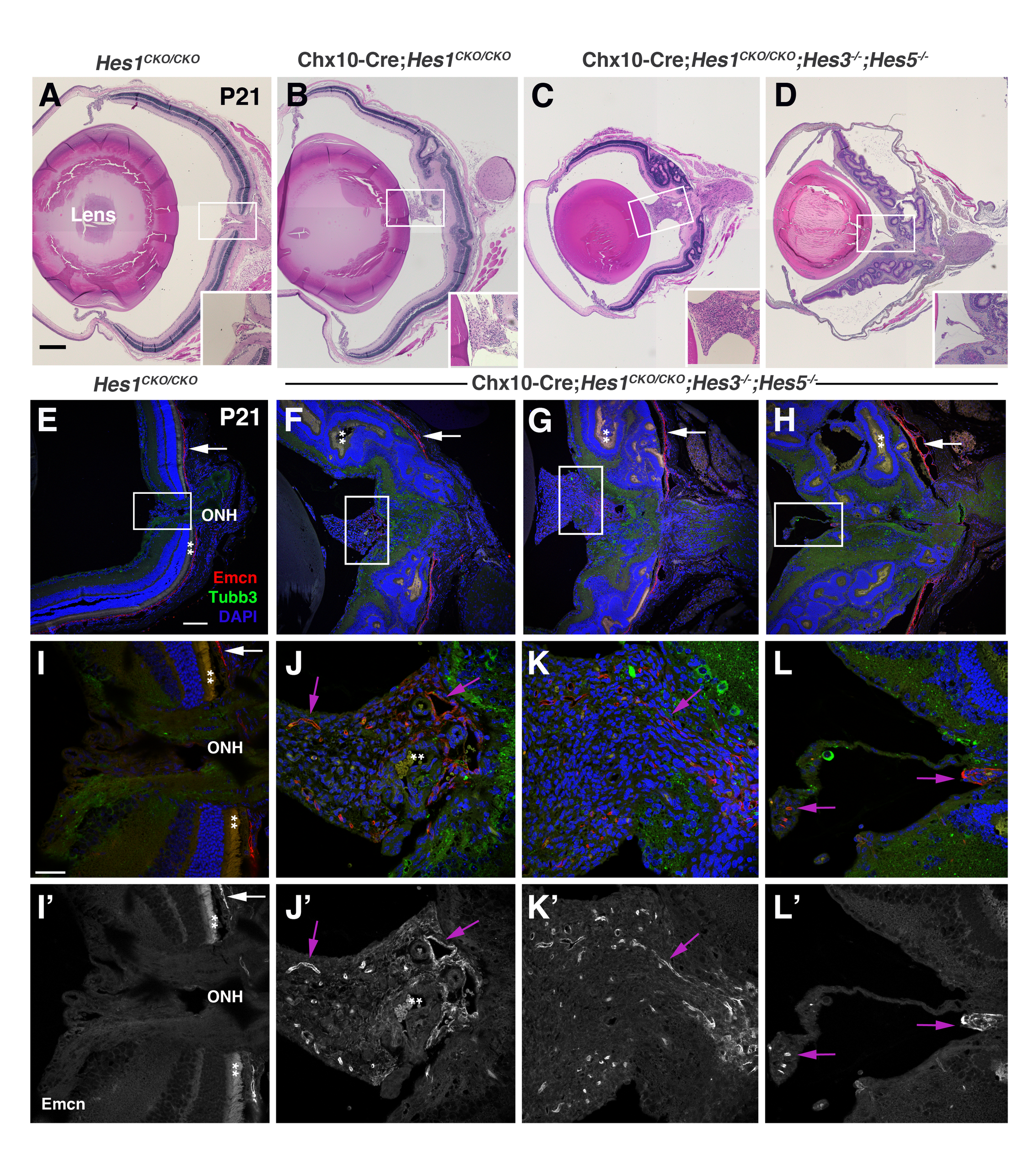

Supplement: S3 Fig — (A-D) H&E staining highlights a range of ocular defects in adult eyes. Boxed areas at higher magnification in inset. (B) Without Hes1 an ectopic vitreal cell mass resides next to the ONH and there are sporadic retinal rosettes. (C-D) Chx10-Cre;HesTKO eyes have more extensive retinal lamination defects and abnormal ONH morphology. (E-L’) Colabeling for Tubb3 (green, neuronal processes) and Endomucin (red, endothelial cells). (I-L’) Higher magnification of boxed areas in E-H. Endomucin labeling of choroid vessels (white arrows) and blood vessels within abnormal vitreal cell masses (pink arrows). This ectopic tissue is largely devoid of Tubb3+ neurons or neural processes. Panels E,I,I’ are of an adjacent section to A; panels G,K,K’ are an adjacent section to C; panels H,L,L’ are an adjacent section to D. Asterisks in I, I’ or J,J’ indicate autofluorescent photoreceptor outer segments or red blood cells within ectopic vessels. Scalebars in A = 200 microns, E,I = 20 microns; n = 3 biologic replicates per genotype. (TIF) [file pgen.1010928.s008.tif]

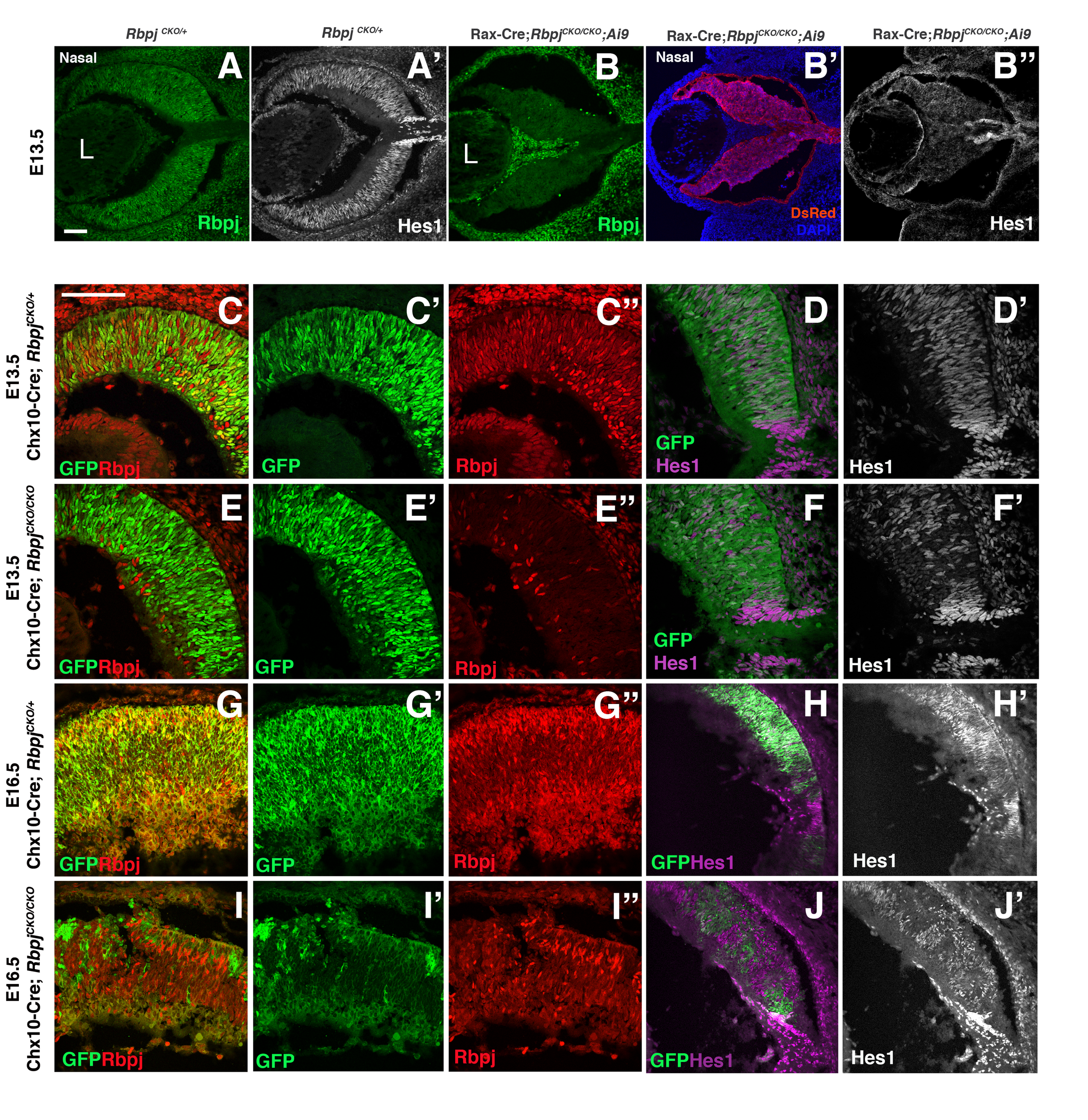

Supplement: S4 Fig — (A-A’) Normal E13.5 expression patterns for Rbpj and Hes1. (B-B") Complete loss of Rbpj in Rax-Cre lineage-marked cells (optic cup, RPE, ONH, OS) in red, see B’. There was also a loss of Hes1 in the cup and RPE, but not in the attenuated ONH/OS (B”). (C-D’) Anti-Rbpj and GFP labeling highlights Chx10-Cre-GFP mosaicism, with scattered GFP-negative retinal cells (red only nuclei in C, pink only in D). Chx10-Cre expression does not spread into the ONH (D). (E-F’) In Chx10-Cre;Rbpj mutant littermates, Rbpj+ cells are dramatically reduced, although the Hes1 retinal domain is less effected (F). Hes1 in the ONH is unaffected in Chx10-Cre animals as expected. (G-H’) At E16, Cre-GFP, Rbpj and Hes1 are normally coexpressed. (I-J’) Proportionally bigger Cre-GFP-neg regions of Chx10-Cre;Rbpj mutant retinas express Rbpj. In J, islands of GFP+ mutant cells are surrounded by Hes1-expressing cells, which either did not undergo Cre recombination or are wild type cells that eventually outcompete and subsequently outnumber the mutant cells. Scalebar in A, C = 50 microns, L = lens in A,B; n = 3 biologic replicates/genotype. (TIF) [file pgen.1010928.s009.tif]

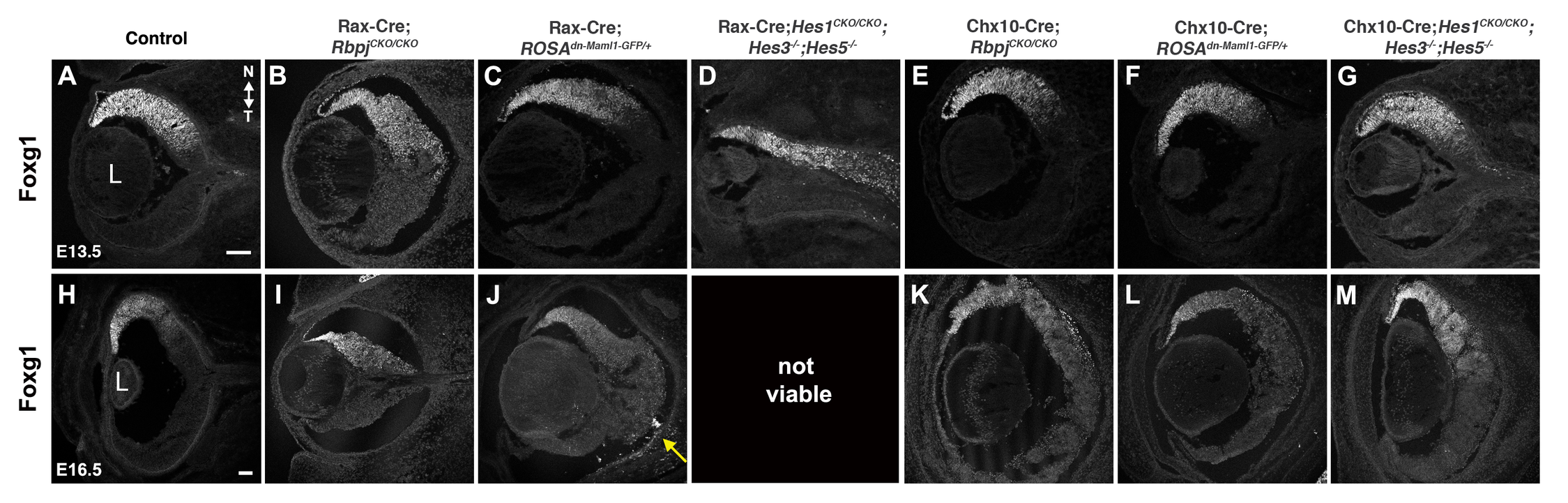

Supplement: S5 Fig — (A-G) At E13.5 Foxg1, in the nasal retina, is properly patterned among nearly all mutants. Only Rax-Cre;HesTKO eyes (D), showed Foxg1 expansion into the optic stalk, consistent with other RPC markers (Figs 1G,3D,3K), where it remained biased to the nasal portion of the retina and optic stalk. (H-M) At E16.5, all mutants have nasally-restricted Foxg1 expression, except Rax-Cre;ROSAdnMAMl1-GFP/+ retinas that have some Foxg1+ nuclei present on the temporal side and within the adjacent subretinal space (arrow in J). All panels oriented nasal up (noted in A) and brain to the right; with L = lens in A,H; scalebar in A, H = 50 microns; n = 3 biologic replicates/genotype. (TIF) [file pgen.1010928.s010.tif]

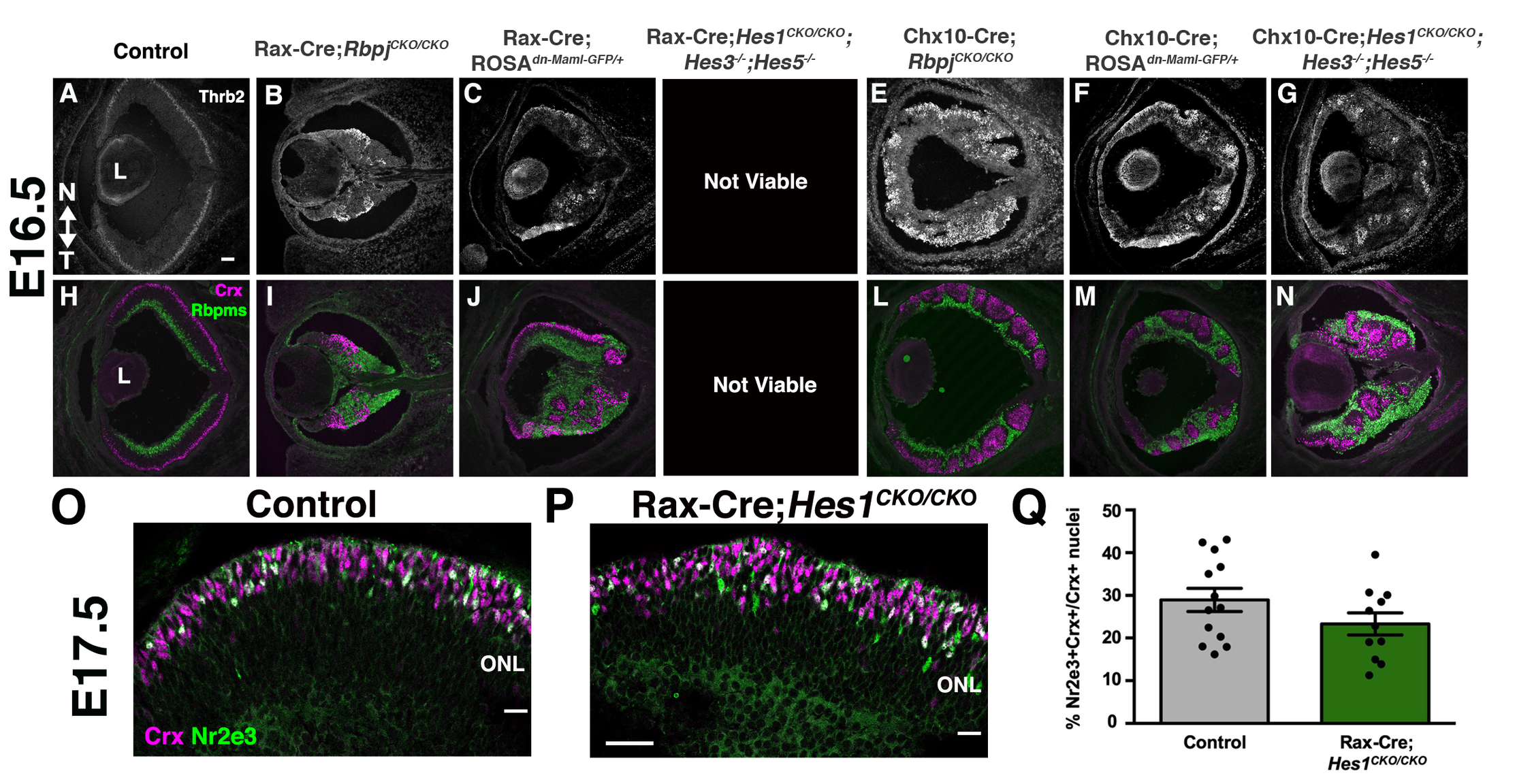

Supplement: S6 Fig — (A-G) Immunostaining for the cone-specific Thrb2 marker at E16.5. (H-N) Crx-Rbpms colabeling of adjacent E16.5 sections highlights the abundance of RGCs and cones relative to other unlabeled cells, as well as photoreceptor rosettes surrounded by RGCs. Panels A-N oriented nasal up, n = ≥3 biological replicates/genotype. (O,P) Crx-Nr2e3 double labeling of E17.5 control and Rax-Cre;Hes1CKO/CKO retinas. (Q) Quantification of colabeled cells within the Crx population indicates no difference in nascent Nr2e3+ rods between genotypes. Panels A-N oriented nasal up (indicated in A), panels O,P oriented scleral up; graphical data in Q represents 13 control and 11 tile scanned composite images from 3 biologic replicates/genotype, displaying individual replicate data points, mean and standard deviation. A student t-test, with unequal variance was used to calculate a p-value in Q. Scalebars in A = 50 microns, P = 20 microns, L = lens in A,H; ONL = outer nuclear layer. (TIF) [file pgen.1010928.s011.tif]
