# Supplementary material for: Notch pathway mutants do not equivalently perturb mouse embryonic retinal development
Source: PLoS Genet. 2023 Sep 26;19(9):e1010928. doi: 10.1371/journal.pgen.1010928 (PMC10522021; doi:10.1371/journal.pgen.1010928)
Supplement: Attachment — Submitted filename: 133380_1_rebuttal_2729441_rxq268.docx [file pgen.1010928.s012.docx]

**Manuscript number:** RC-2023-01831

**Corresponding author(s):** Nadean L. Brown

## 1. General Statements

We will not repeat the goals of the study here, they are articulated below, in the Abstract and in the author summary. We appreciate the points raised by all reviewers, which prompted us to produce a much clearer revised manuscript. In particular, a request for quantification of retinal phenotypes induced us to make novel findings about the role of Notch signaling in neurogenesis.

## 2. Point-by-point description of the revisions

Note to editors: Reviewer comments are in black font, our detailed responses that also describe additional experiments and changes to figures and text are shown in blue font.

RESPONSE TO REVIEW COMMONS REVIEWERS:

We thank the reviewers for their insights and comments on this manuscript. Specific responses to reviewer concerns are detailed below. We made a couple of significant changes based on the feedback. First, we performed more experiments to increase biologic replicates and then quantified image data for multiple figures. The new quantitative information added to Figure 3 fully supports our original conclusions about changes to the ONH in *Hes-TKO* mutants. The quantification of Atoh7, Otx2, Rbpms and Crx expressing cells among the different genotypes revealed interesting differences in Notch intracellular gene requirements for both RGC and cone development. The most startling outcome is that changes in both cell types correlate with significant changes in Otx2, but not Atoh7. This singular finding suggests interesting future work is needed, well beyond the scope of this paper about the molecular mechanisms underlying these cell fates. Second, our data presentation was reorganized with new information added to Fig 1 that clarifies the relationships between Hes1, Hes5, Foxg1 and Pax2; old Figs 6 & 7 about neurogenesis were merged; and some data moved to new Suppl Figs 2 and 5. The numbering for multiple figures changed and a new summary model (now Fig 8) is provided. In addition, the manuscript was completely rewritten to improve clarity. We hope this revised manuscript is acceptable for publication.

Reviewer #1 Summary:

In this study, the authors employed an impressive set of mouse mutant or Cre lines to investigate the complexity of Notch signaling across different stages of retinal development. These comprehensive analyses led to two main findings: 1. Sustained hes1 in the OHS/OS is Notch-independent; 2. Rbpj and Hes1 exhibited opposing roles in cone photoreceptor development. Although the study is potentially interesting, the current manuscript needs the essential research background and quantification, a lack of which significantly reduced the clarity of the manuscript and the credibility of the major conclusions. Also, how the authors organized the results is quite confusing, making the manuscript very difficult to follow.

Response: We agree with all reviewers concerning incomplete quantification of the data. We directly addressed this shortcoming in revised Figs 3 and 6 (the latter combines old Figs 6 +7). To do this, we repeated some IHC experiments to add more replicates and reorganized all of the neurogenesis phenotypic data figures. Our quantifications uncovered several surprising outcomes that clarify our model. For these reasons, the manuscript was exhaustively rewritten. We merged E13 neurogenesis data into revised Figure 6 and moved the most relevant E16 analyses to new supplemental data Fig 5. All changes made should make the paper easier to understand for retinal development, neurogenesis, and Notch pathway aficionados, in addition to readers lacking such expertise.

Major comments: 
1. The authors needed to make the quantification for many analyses to strengthen the conclusions, such as Fig. 1F, 1G, and etc.

Response: We quantified optic nerve head (ONL) immunohistochemistry data in the revised Fig 3. We also quantified neurogenesis markers Atoh7, Otx2, Rbpms (RGCs), and Crx at E13 in revised Fig 6 (former Figs 6 and 7). Older stages were moved to a new Suppl Fig 5.

Respectfully, *Hes5* mRNA expression in old Fig 1F and 1G shows that *Hes5*, like other retinal progenitor cell (RPC) markers, expanded in Rax-Cre deletion but not Chx10-Cre deletion conditions. This is analogous to Pax6 and Rax expansion in Rax-Cre;*Hes1 CKO* eyes and *Pax2* mutants (doi: 10.1523/JNEUROSCI.2327-19.2020) (1). In revised Fig 1, we now show analogous expansion of *Hes5* mRNA in *Pax2* mutant retinas (compare Figs 1F-1I). Because *Hes5* RNA in situ hybridization experiments are nonquantitative, we do not discuss the possibility of *Hes5* mRNA level changes in labeled cells.

2. The authors reported many exciting results. However, further mechanistic insights are largely missing. They may focus on one of these exciting findings and give some mechanistic insights. For example, hes1 suppresses hes5 expression as the ONH boundary forms; hes1 expression in the ONH is Notch independent; differential influences of Rbpj and Hes1 on cone development. It is better for the authors to select one of these exciting findings and provide a deeper mechanistic study.

Response: This revision brings fresh focus to Notch regulation of RGC and photoreceptor development, particularly differential influences for *Rbpj* versus *Hes1*. We also better support our interpretation of image data in Fig 1. We include new data about the spatial relationships between Hes5-GFP/Pax2 and Hes5-GFP/Foxg1. In summary, we find that as Pax2 becomes restricted to the nasal optic cup prior to the onset of RGC genesis, it becomes mutually exclusive with Hes5-GFP, at the same time that Hes5-GFP+ cells coexpress Hes1. This is consistent with Hes1 indirectly regulating Hes5-GFP as a marker of neurogenic RPCs at the forming ONH. Furthermore, it emphasizes the importance of genetically teasing apart the separate and potentially compensatory roles for Hes1 versus Hes5 undertaken here. These relationships remain poorly resolved during vertebrate CNS development.

3. Some analyses lack an explanation of the rationale. For example, "To understand if the loss of multiple Hes genes is more catastrophic than Hes1 alone..."(PAGE 7). Please explain its significance.

Response: We assume the reviewer is referring to the first sentence of the last paragraph on this page. We analyzed *Hes* triple mutant mice (TKO) to understand if removing multiple *Hes* genes reveals redundant functions. This is an open question, given that Hes1 is expressed in the ONH/OS, which is normally devoid of Hes5 by the time retinal neurogenesis begins. These questions have only been explored in a handful of tissues throughout the body. Also see response to point 2 above. In general, we have expanded the rationale for all of the experiments throughout the revised manuscript.

Significance: In general, many results are quite interesting. However, the significance of these findings is largely hampered in the following aspects: 1. The authors were unable to provide the sufficient research contexts that are essential for understanding many results.2. Many conclusions were solely based on descriptive images but lacked statistical quantification, which significantly weakened many conclusions. 3. Many interesting findings are quite descriptive, and some mechanistic understandings of one of these exciting findings will be beneficial to improve the focus and significance of the study.  Current format of the manuscript fits more specialized audience.

Response: During *in vivo* development, we wished to understand which particular Notch pathway genes can interact in a Notch-dependent versus a Notch-independent manner. Genetic (phenotypic) studies produce extremely rigorous datasets, in our opinion. This revision now extensively quantifies key findings. Here we dissected the "receipt" of a Notch signal by identically testing the functional requirements of particular pathway members. For Mastermind (Maml), there are 3 paralogues, double mutants for *Maml1* and *Maml3* are early lethal, and no floxed alleles exist, so it was logical to employ the ROSA-dnMaml mouse strain, particularly since it has been discussed throughout the Notch literature as "analogous" to removing either a Notch receptor or Rbpj. Our finding that the dnMAML allele does not function like a Rbpj null in the retina is important for researchers in the broad Notch field to consider when designing and interpreting experiments.

Reviewer #2: 
Hes genes are effectors of the Notch signaling pathway but can also act down-stream of other signaling cascades. In this manuscript the authors attempt to address the complexity of Hes effectors during optic cup development and retinal neurogenesis. To do so, they compared optic cup patterning and retinal neurogenesis in seven germline or conditional mutant mouse embryos generated with two spatio-temporally distinct Cre drivers. These lines allowed for the analysis of the consequences of perturbing the Notch ternary complex and multiple Hes genes alone or in combination. The authors show that the optic disc/nerve head is regulated by Notch independent Hes1 function. They also confirm that perturbation of Notch signaling interferes with cell proliferation enhancing the production of differentiated ganglion cells, whereas photoreceptor genesis requires both Rbpj and Hes1 with Notch dependent and independent mechanisms. 
This is a rather complex study that dissects further the role of the Notch pathway and Hes proteins during eye development, a topic that has been addressed in many previous studies but perhaps not with the details that the authors have used here. In this respect, this study adds to current literature but will likely be of interest to retina aficionados. The manuscript reads well and the figures are of very good quality. However, many of the statements are based on qualitative rather than on quantitative analysis. This should be, at least in some cases, remediated, despite the effort that this may require given the number of mouse lines used in the study.

Response: As described in the response to Reviewer 1, we agree and present considerably more quantification data. We extensively reorganized and rewrote this manuscript to emphasize that Hes1 in the ONH/OS is fully Notch-independent and highlight branchpoints in Notch-dependent signaling, for Rbpj versus Hes,1 during early retinal neurogenesis. It is too simplistic that the ternary complex (Rbpj-NICD-Maml) simply activates Hes1 (and/or multiple Hes genes) to regulate downstream signaling targets. This paradigm has been portrayed in the literature numerous times for many processes throughout vertebrate development, homeostasis or relative to particular diseases. By focusing on one tissue and a narrow window of development, our phenotypic studies delved more deeply to show the greater complexity and molecular cross-talk that we think underlie the modulation of signaling levels with in vivo context. Thus, our results are of broad interest and impact to the greater Notch field.

1. The title is somewhat misleading. The authors have explored mostly the role of Hes1, 3 and5. Although these are Notch effectors, there is already evidence that they participate in other pathways This is confirmed by the data present here. I would suggest to eliminate Notch from the title and use instead "Hes" to better reflect the findings. Furthermore, it is unclear why there is a reference to "mutations" or what are the Notch branchpoints to which the authors refer at the beginning of the discussion.

Response: We appreciate the reviewer’s viewpoint but disagree this paper is mostly about *Hes* genes, as there is a critical direct, comparable evaluation with *Rbpj* and dn-Maml. Direct comparison of 7 genotypes highlights where each pathway member exhibits idiosyncratic phenotypes. We are striving for a clear, simple title about a very complex topic, involving the in vivo genetic dissection of a signaling pathway. We modified the title to: "Notch pathway mutations do not equivalently perturb mouse embryonic retinal development "

2. "Although the Pax6-Pax2 boundary is intact in Rax-Cre;RbpjCKO/CKO eyes, ONH shape was attenuated compared to controls (Fig 3I)". This statement is arguable as the difference seems subtle. Perhaps some kind of quantification would help.

Response: We quantified Pax2+ cells (ONH domain) using the adjacent proximal terminus of the retinal pigmented epithelium (RPE) to indicate a transition from ONH to optic stalk (OS). We also quantified the number of Pax2+Pax6+ double positive cells where the 2 domains abut (boundary cells). Some higher magnification examples are now provided in Fig 3H';3K';3N'. Grossly, the imaging data support that the Pax2+ ONH is expanded in Chx10-Cre;TKO eyes, while boundary cells are most affected in Rax-Cre;*HesTKO* eyes, due to an expansion of retinal tissue. This is supported by our quantitative data (Fig 3O,3P). We observed even in controls that Pax2-expressing cells show some numerical variability. We attributed this to the position of the section through the ONH, which is a 3-dimsenional ring (torus). Therefore, we quantified additional wild-type controls and mutant samples in the new Fig 3O,3P graphs, improving statistical power, and allowing us to detect quantitative differences.

3. Page 12 first paragraph. "....but all other genotypes were unaffected". This statement is unclear. All lines in which the Rax-Cre has been used seem to have an increased number of apoptotic cells. This should be better explained

Response: Respectfully, only one genotype, Rax-Cre;*Rbpj* mutants contain a *statistically significant* increase in apoptotic cells (Fig 5P). This is demonstrated by one-way ANOVA analyses that included all pairwise comparisons. To ensure that the quantification was not misleading due to changes in tissue morphology, data in Figs 5, 6, and 7 were normalized to optic cup area. The area was traced in FIJI, creating a polygon whose area was determined in square microns. For every section image, the marker+ cells were divided by the square micron area of the retina (excluding the opening for the optic nerve). Such a method is critical for comparison across this allelic series, given the morphologic changes, differences in cell clustering where rosettes form, and reduced proliferation whenever Notch signaling is lost or reduced.

4. Page 12, end of second paragraph: "E13.5 Chx10-Cre;HesTKO eyes had a milder RGC phenotype (Figs 6G, 6N, 6U), but all other mutants were unaffected (Figs 6E, 6F, 6L, 6M, 6S, 6T). This statement is also rather subjective. The phenotype of Chx10-Cre;HesTKO is quite strong and the other mutants seem to have a phenotype. Some quantifications here will help.

Response: We agree and provide quantification for both Atoh7 and Rbpms positive cells in the revised Figure 6. This is now in the same figure with quantification of Otx2+, Otx2+Atoh7+ and Crx+ cells. The reviewer is correct that both ROSA-dnMaml and both *HesTKO* mutants have a statistically significant increase in RGCs. Surprisingly, neither of the *Rbpj CKO* mutants have this outcome (Fig 6Y).

5. Page 13, toward the bottom..."...but noted that Chx10-Cre RbpjCKO/CKO eyes were not different from controls (Figs 7E, 7AA)". Again, this statement is questionable as staining for both CRX and Rbpms seem reduced as compared to controls as quantifications in 7AA seems also to indicate (about half?). Did the authors calculate whether there is a statistical difference between controls and Chx10-Cre RbpjCKO/CKO ?

Response: Rbpms+ RGCs and Crx+ photoreceptor precursors were colabeled and quantified on sections for all genotypes. All counts were normalized to area as described above. Upon quantification and ANOVA with pairwise comparisons, there was no statistical difference in Crx+ or Rbpms+ cells between control and Chx10-Cre;*Rbpj* mutants (new Fig 6Y and Z).

6. In Fig 7CC the authors should make the effort of including at least one additional sample, 2 biological replicates seem insufficient to draw a conclusion.

Response: The Rax-Cre;*Hes1*CKO/+ X *Hes1*CKO/CKO matings stopped producing litters in late 2022. While this manuscript was out for review, we obtained younger mice, from which new control and Rax-Cre; *Hes1* mutant littermates were collected, stained, imaged and quantified. Upon adding samples, we found that the outcome was unchanged, but the data better support the lack of a statistical difference in rods between genotypes at E17. These data were moved to revised Suppl Fig 5.

Significance: This is a rather complex study that dissects further the role of the Notch pathway and Hes proteins during eye development, a topic that has been addressed in many previous studies but perhaps not with the details that the authors have used here. In this respect, this study adds to current literature but will likely be of interest to retina aficionados. The manuscript reads well and the figures are of very good quality. However, many of the statements are based on qualitative rather than on quantitative analysis. This should be, at least in some cases, remediated, despite the effort that this may require given the number of mouse lines used in the study.

Response: To increase the impact of our manuscript, we quantified all markers except Tubb3, since its localization in cell bodies and axons make it impossible to assign to individual cells. We feel that this additional quantification strongly improves the quality of our findings and allowed us to make well-supported and novel conclusions. While we certainly believe that the retinal development community will find this paper of interest, it will also be of value to the broader Notch pathway scientific community. In this manuscript, we simultaneously compared phenotypes for Notch pathway genes in signal receiving cells. We could find essentially no studies like this for the mouse CNS and only a few from Raphael Kopan's lab about the kidney and immune system. Interestingly, one of us (NLB) is a coauthor on a recent paper about Notch signaling in the cortex, in which ROSA-dnMaml behaves analogously to *Notch1CKO* or *RbpjCKO*. This emphasizes that findings in one organ may not recapitulate the "rules" for this pathway for other cell types or tissues (doi: 10.1242/dev.201408)(2). Deeper understanding of how the Notch pathway in the retina functions, analogously or differently, is important. We feel our revised study advances when and where there are "branchpoints" in canonical signaling that may be overlooked in other developing tissues and organs.

Reviewer #3: 
I have reviewed a manuscript submitted by Bosze et al., which is entitled "Not all Notch pathway mutations are equal in the embryonic mouse retina". The authors focused on Notch signaling pathway. Notch signaling is deeply conserved across vertebrate and invertebrate animal species: in general, two transmembrane proteins, Delta and Notch, interact as a ligand and a receptor, respectively, which induces proteolytic cleavage of Notch receptors to generate Notch intracellular domain (NICD). NICD is translocated into nucleus, then forms the transcription factor complex including Rbpj (also referred to as CBF1) and Mastermind-like (Maml), and activates the transcription of Hes family transcription factors. Three Hes proteins, Hes1, 3, and 5, are important for nervous system development. In the vertebrate developing retina, these Hes proteins inhibit neurogenesis to maintain a pool of neural progenitor cells. In addition to their primary role in neurogenesis, the authors recently reported that Hes1 promotes cone photoreceptor differentiation. In the later stages of development, Hes proteins also promote Müller glial differentiation. In addition, Hes1 is highly expressed in the boundary between the neural retina and optic stalk and required for this boundary maintenance. 
To understand precise regulation of Notch component-mediated signaling network for retinal neurogenesis and cell differentiation, the authors compared retinal phenotypes in the knockdown of three Notch pathway components, that is (1) Hes1/3/5 cTKO, (2) Rbpj KO, and (3) dominant-negative Maml (dnMaml) overexpression, under the control of two Cre derivers; Rax-Cre and Chx10-Cre. 
First, the authors found that Hes1 expression in the boundary between optic stalk and neural retina is lost in Rax-Cre; Hes1/3/5 cTKO, but still retained in Rax-Cre; Rbpj KO and Rax-Cre; dnMaml overexpression, suggesting that Delta-Notch interaction is not required for Hes1 expression in the boundary between optic stalk and neural retina. Furthermore, Hes1 expressing boundary region expands distally at the expense of the neural retina in Chx10-Cre; Hes1/3/5 cTKO. Maintenance of ccd2 expression in this expanded boundary area suggests that Hes1 normally maintains a proliferative state in the optic stalk, which may allow these cells to differentiate into astrocyte in later stages. 
Second, in addition to precocious RGC differentiation in all the Notch component KO, the authors found that, as compared with wild-type, cone and rod photoreceptor genesis is highly enhanced in Rax-Cre; Rbpj KO and Rax-Cre; dnMaml overexpression and mildly enhanced in Chx10-Cre; dnMaml overexpression. On the other hand, in Rax-Cre; Hes1/3/5 cTKO, cone and rod photoreceptor genesis is not enhanced but similar to wild-type level. Since the authors previously reported that cone genesis is reduced in Rax-Cre; Hes1 cKO and Chx10-Cre; Hes1 cKO, so Rax-Cre; Hes1/3/5 cTKO may rescue decrease in cone genesis in single Hes1 cKO. The authors raise the possibility that elevated Hes5 expression in single Hes1 cKO may suppress cone photoreceptor genesis. The authors also found that amacrine cell genesis is significantly suppressed in Rax-Cre; Rbpj KO but not changed in Rax-Cre; dnMaml overexpression and Rax-Cre; Hes1/3/5 cTKO, suggesting that Rbpj is specifically required for amacrine cell genesis. From these observations, the authors propose that there are at least two branchpoints for photoreceptor and amacrine cell genesis in Notch component-mediated signaling network. 
Their findings are very interesting and provide some new insight on how Notch signaling components are integrated into other signaling pathways and promote to generate diverse but well-balanced retinal cell-types during retinal neurogenesis and cell differentiation, in addition to conventional classic view of Notch signaling pathway. However, one weak point is that, although the authors figured out what kinds of phenotypic difference appear in the KO retinas between these Notch components, the research result is descriptive and less analytical. Most of their conclusions may be supported by their previous works or others; it is still hypothetical. So, it is important to show more analytical data to support their interpretation and more clearly show what is new conceptual advance for Notch signaling pathways. 

For example, sustained Hes1 expression in the boundary region between optic stalk and neural retina may be reminiscent to brain isthmus situation. I would like to request the authors to show more direct evidence that Hes1 regulation in optic stalk/retina boundary is independent of Delta-Notch interaction. One possible experiment is whether DAPT treatment phenocopies Rax-Cre; Rbpj KO and Rax-Cre; dnMaml overexpression (Hes1 in optic stalk boundary is normal?).

Response: Usage of the gamma secretase inhibitor DAPT is an interesting experiment as it can phenocopy the loss of Notch signaling in developing tissues. However, the reviewer's proposed DAPT experiment is problematic for two major reasons. First, DAPT blocks the gamma secretase complex, which has more than 90 protein targets in the cell membrane (3). Therefore, DAPT may not be informative for *Hes1* regulation given the myriad of expected off-target effects. Second, it would be difficult to treat embryos at the relevant stages with DAPT. Injections into pregnant mice are lethal and we cannot localize drug to the relevant area during in vivo development. Our direct phenotypic comparisons with two Cre drivers strongly indicate that *Hes1* is independent of canonical Notch signaling in the developing optic stalk.

We include a figure for the reviewer showing anti-Hes1 immunolabeling of E13.5 Rax-Cre;*Notch1CKO/CKO* (n=2) and E13.5 Rax-Cre;*Notch2CKO/CKO* eyes (n=3). The *Notch1* mutant lost oscillating Hes1 expression in retinal progenitors, but the uniform Hes1 ONH domain remains. Interestingly, the *Notch2* mutant had essentially no effect on Hes1 (oscillating or sustained), or *Hes5* mRNA expression. A *Notch2* RNA in situ hybridization demonstrates that *Notch2* mRNA was lost in the E13 optic cup and RPE (Rax-Cre expressing tissues). These data emphasize: A) the Notch1-specific dependency of oscillating Hes1 expression in retinal progenitors is absent from the ONH; B) although coexpressed in the same tissue, Notch receptors have unequal activities.

Does Rax-Cre; Rbpj KO; Hes1-cKO phenocopy Rax-Cre; Hes1-cKO (or Rax-Cre; Hes1/3/5 cTKO)?

Response: This is a good question! The first author tried very hard to produce Rax-Cre; *Rbpj CKO*;*Hes1 CKO* double mutant embryos. However, these progeny could not be recovered from E10-E13 embryos, despite collecting more than 10 litters. Thus, it is likely that this genotype is lethal before eye formation.

Could the authors identify an enhancer element that drives Hes1 transcription in optic stalk/retina boundary, which should be not overlapped with that of NICD/ Rbpj binding motif? Such additional evidence will make their conclusion more convincing.

Response: Another interesting question. We have been working for >3 years on Hes1 cis regulatory enhancers, but the pandemic greatly delayed progress. The proximal *Hes1* 600bp upstream region is a generic enhancer that contains Hes1 binding sites for repressing its own expression (4) and has a pair of Rbpj consensus sites for Notch ternary complex activation of Hes1 expression (5,6). Nearby is a binding site occupied by Gli2 in the E16 mouse retina (7). Recently, it was shown that Ikzf4 binds slightly farther away (8). The upstream 1.8 kb region (including the 600bp just described) can drive destabilized GFP or dsRed reporters in early postnatal retinal explants (9). However, this sequence was used to make and analyze a classic Hes1-GFP transgenic reporter mouse, in which GFP was **not** expressed in the early embryonic mouse optic vesicle or cup (10). Therefore, any early eye-specific enhancer(s) are located farther upstream, in an intron, or downstream (or combination thereof). Public domain epigenetic and chromatin accessibility datasets support this idea. Identifying the gene regulatory logic for Hes1 expression in the eye will be an exciting future story, well beyond this manuscript. We are excited to use live imaging of enhancer reporters to discern oscillating versus sustained activity patterns during early ocular development.

Regarding the conclusion on new branchpoints on photoreceptor and amacrine cell genesis, a model shown in Figure 9 is still hypothetical. Figure 9B indicate a model in which the increase of Otx2+ cells and Crx+ cells in Rax-Cre; Rbpj KO is mediated by Hes1, which is presumed to be activated in Notch-independent signaling. However, Hes1 expression in the neural retina is markedly reduced in Rax-Cre; Rbpj KO (Fig. 2I), which does not fit in with the model.

Response: We removed Fig 9B and now present new models about the Notch-dependent versus -independent roles for both Rbpj and Hes1. The new summary is Fig 8.

So, I would like to request the authors to examine whether the increase of Otx2+ cells and Crx+ cells in Rax-Cre; Rbpj KO, (or Rax-Cre; dnMaml overexpression and Chx10-Cre; dnMaml overexpression) is inhibited by Hes1 KO.

Response: If we understand this correctly, it would mean generating double mutants, some of which we determined are not viable (see the response above, and Suppl Table 2). Given there is only a partial knockdown of Hes1 or Hes5 in either dnMaml mutant we do not believe repeating this in the *Hes1 CKO* genetic background to be informative and it would take 3 generations to perform.

Second, the authors concluded that both cone and rod genesis are enhanced in Rax-Cre; Rbpj KO by showing the data on Crx/Nr2e3 labeling in Rax-Cre; Hes1 cKO in Fig. 7BB. However, as the authors mentioned in the manuscript, Hes5 expression is elevated in Rax-Cre; Hes1 cKO (Fig. 1G). So, since Rax-Cre; Hes1 cKO has residual Hes activity in the retina, Fig. 7BB should be replaced with labeling of Crx/Nr2e3 in Rax-Cre; Hes1/3/5 cTKO.

Response: Unfortunately, Rax-Cre;*HesTKO* embryos do not live past E13 (Suppl Table 2). Thus, we cannot evaluate rods, whose genesis starts around E13.5. Revised Fig 1G shows the *Hes5* domain is shifted with the expansion of retinal tissue in E13.5 *Hes1* single mutants, but importantly, also analogously shifted in *Pax2* mutants (Fig 1H). We do not conclude that mRNA levels are "elevated" since mRNA in situ hybridization is not a quantitative technique.

Our initial examination of rods in E17 Rax-Cre;*Hes1* CKO mutants tested the idea of a fate shift from cones to rods. However, deeper quantification (Suppl Fig 5) do not support such a fate change.

Furthermore, possibly, it is best to examine labeling of the retinas of Rax-Cre; Rbpj KO with rod and cone-specific markers and confirm that the number of both rods and cones is significantly increased. Third, as for defects in amacrine cells genesis in Rax-Cre; Rbpj KO, I would like to request the authors to show the data on Crx10-Cre; Rbpj KO. Although Rbpj KO is mosaic in Crx10-Cre; Rbpj KO, we can distinct Rbpj KO cells by GFP expression (Fig. S2C, C', C'). So, the authors can confirm that amacrine cell genesis is inhibited in a cell-autonomous manner in Crx10-Cre; Rbpj KO retinas but not in Crx10-Cre; dnMaml overexpression. Addition of such data will make the authors' conclusion is more convincing.

Response: Suppl Table 1 lists multiple references (two from the NLB lab) that demonstrated both a rod and cone increase in *Rbpj* loss-of-function conditions. Chx10;*Rbpj* CKO animals were evaluated by Zheng et al., who showed an amacrine loss phenotype in these mutants (11). This is equivalent to what we see in our Rax-Cre;*Rbpj* CKO data, but without the complications of Chx10 mosaic Cre expression upon *Rbpj* deletion.

Other comments: 
1) Title of this manuscript is "Not all Notch pathway mutations are equal in the embryonic mouse retina". However, this title is quite obscure in what is research advancement of their findings. I suggest the authors to include more concrete and conclusive sentence in the title, for example "Hes and Rbpj differentially promotes retina/optic stalk boundary maintenance and photoreceptor genesis, in parallel with neurogenic inhibition by Notch signaling pathway".

Response: We appreciate the reviewer's perspective. We are striving for a relatively simple title about a very complex topic, involving the in vivo genetic dissection of a signaling pathway. We modified the title to "Notch pathway mutations do not equivalently perturb mouse embryonic retinal development ".

2) The "Results" section is a bit difficult to follow logics without detailed knowledge on roles of Notch signaling in mouse retinal development. I suggest the authors to improve a writing style of "Results" section for readers without such detailed knowledge on mouse Notch mutant phenotypes to follow logical flow more easily. There are many additional descriptions on research background before start to mention results. Such introductory sentences should be moved to the "Introduction" section, by which logical flow in the Results section should be simpler. In addition, the authors should show a concrete question at the beginning of each result subsection. Furthermore, the authors sometimes jump over from one result subsection and suddenly move to cite another figure panel in a far ahead subsection whose data has not been explained. Such a back-and-forth citation of figure data generally makes it difficult to follow logical flow.

Response: We now present a considerable amount of new quantified data, reorganized multiple figures, and extensively rewrote the paper. We significantly revised the summary figure to improve clarity. In addition, Suppl Table 1 provides a wealth of background information to orient the reader on this topic. We feel that this extensive revision has greatly improved the quality, logical flow, and readability of the manuscript.

3) In addition, figure configuration is not well organized. Each figure compared some particular marker expression in wild-type, Rax-Cre; HesTKO, Rax-Cre; Rbpj cKO, Rax-Cre; dn-Maml-GFP, Chx10-Cre; HesTKO, Chx10-Cre; Rbpj cKO, Chx10-Cre; dn-Maml-GFP. For example, Fig. 2 shows Hes1 for inhibition of neurogenesis, Fig. 3 shows Vsx2; Mitf and Pax2; Pax6 for retinal pigmented epithelium and optic stalk, Fig. 6 shows Atoh7, Rbpms, and Tubb3 for retinal ganglion cells. Fig. 7 shows Crx, Otx2, and Thrb2 for photoreceptor differentiation. Fig. 8 shows Prdm1, and Ptf1a for photoreceptors and amacrine cells. Although this figure configuration is convenient to show phenotypic difference between different genetic mutations, it is difficult to know how each differentiation steps are spatially and temporally coordinated during development. At least, I recommend the authors to show one summary figure, which shows spatio-temporal expression profile of retinal markers in wild-type mouse retinas.

Response: We recognize this point and completely reorganized and combined Figs 6 and 7 to improve clarity. New Figure 6 presents E13 quantification for Atoh7, Otx2, Atoh7/Otx2, Rbpms and Crx expressing retinal populations. E16-E17 data were condensed and moved to a new Suppl Fig 5.

4a) Page 7, line 7-10 "With earlier deletion using Rax-Cre, hes5 mRNA abnormally extended into the optic stalk": I wonder how the authors define the optic stalk. It is likely that optic stalk area (Pax2+, Vax1+ area) is shifted to more proximal (depart from the optic cup and move toward the brain), and neural retina is expanded accordingly (Fig. 4B, 4F), resulting in expansion of hes5 expression. Thus, it may be better to mention that optic stalk/neural retina boundary is abnormally shifted toward the brain.

Response: The retina, including the optic nerve head, ends where the adjacent RPE terminates. This is conspicuous morphologically in our sections. We also defined this by colabeling for Pax2 and Pax6, which is now quantified in revised Fig 3. To clarify this further, we added the words " in all panels the brain is to the right" in the Fig 4 legend.

4b) Page 8, line 14-15, "ONH/OS cells still express it (Hes1), demonstrating that sustained Hes1 is independent of Notch": I presume that Cre-Rax drives Cre in neural retina as well as optic stalk and pigmented epithelium. However, it is likely that Rbpj is not expressed in optic stalk/neural retina boundary area in wild type (Fig. S2A). No expression of Rbpj in optic stalk/neural retina boundary may support that Hes1 expression in this boundary area is Notch-independent. However, Rbpj expression is retained in some vitreal cells near optic nerve head in Rax-Cre; Rbpj-CKO retinas (Fig. S2B). What are these Rbpj+ cells? I would like to request the authors to confirm that Rbpj expression is completely absent in both neural retina and optic stalk in Rax-Cre; Rbpj-CKO mice. Otherwise, this conclusion is still not fully supported.

Response: We show the Rax-Cre lineage in Suppl Fig 2 via the Ai9 (tomato) reporter. The results are striking, with all of the optic cup derivatives (retina, RPE, ONH, optic stalk, and presumptive ciliary tissue and iris) being tomato positive, while the well-described population of vascular cells in the hyaloid space lack tomato expression. Furthermore, our figure shows that Rbpj expression is only absent from the optic cup derivates, rather than the vascular structures in the vitreous. Vascular cells also depend on the Notch pathway and express Rbpj. Based on considerable evidence from the literature and our lineage experiments, the population of cells the reviewer highlights represents the hyaloid vasculature and associated cell types. It does not represent any population that derives from neuroectoderm.

4c) Page 9, line 16-18, "Foxg1 had spread into the nasal optic stalk": Is Foxg1 expanded nasal area really "OS" rather than expanded retina? I suggest the authors to confirm molecular markers Pax2 expression is overlapped with Foxg1. Otherwise, it is difficult to conclude that foxg1 is expanded into the optic stalk territory, because foxg1 is normally a marker of retina. Indeed, Fig. 3K shows pax2 expression is shifted into more inside towards the brain, suggesting that neural retina is expanded. Please explain the situation.

Response: Foxg1 (*BF-1*) mRNA and protein are found in the nasal retina and are expressed in other brain tissues. Multiple studies show Foxg1 in the nasal side of the E10 optic cup/retina/optic stalk and developing hypothalamus (for example, top row, adjacent figure from Smith et al., 2017 (12) with *Foxg1* mRNA in purple. Also see our new manuscript panel Fig 1C. We include here for the reviewer images of E13 ocular cryosections colabeled for Foxg1 and Pax2, highlighting their relationship in the retina, optic stalk and adjacent forming hypothalamus. On page 9 the text now reads "At E13.5 Rax-Cre;*Hes^TKO^* eyes, the Foxg1 nasal retinal domain was contiguous with the nasal optic stalk (Suppl Fig 4D). This is reminiscent of younger stages (Fig 1C), since normally at E13.5, Foxg1 in the nasal optic cup/retina is separated from expression in the ONH/OS (Suppl Fig 4A). Based on the expansion of Pax6, Vsx2 and *Hes5* RPC domains into the optic stalk, we conclude that the change in Foxg1 similarly reflects an extension of retinal tissue."

4d) Page 10, line 4-5, In Rax-Cre; Hes1/3/5 cTKO eye, this tissue (RPE) extended into the optic stalk": This description seems to be incorrect. A part of Pax2 area, which is adjacent to the neural retina, contacts with RPE in wild type (Fig. 3AH), so most of RPE covers the neural retina even in Fig. 3DK.

Response: We disagree with the reviewer’s interpretation. Fig 3D shows Mitf labeling of RPE nuclei. Figure 3K shows the adjacent section labeled with Pax2 and Pax6 (labels both retina and RPE). As the retina extended "towards the brain", the RPE analogously extends and surrounds the retinal domain. We also added higher magnification data panels 3H, 3K and 3N, showing merged and single channels.

4e) Page 10, line 22-23, "For Chk10-Cre; Hes1/3/5 cTKO, there was a unique presence of ectopic Pax2 within the retinal territories": I wonder if this description is correct. I suspect that proliferative Pax2+ cells expand into regressing territory of Hes KO retinal cells, which undergo precocious neurogenesis and lose proliferative activity, in Chk10-Cre; HesTKO. In this case, it is possible that the Pax2/Pax6 interface may be maintained. Please show red and green channel panels for Fig. 3N to confirm that there is ectopic pax2 and pax6 double positive cells.

Response: New quantification in revised Fig 3 (see panels O,P) fully supports our original conclusion. Only Chx10-Cre;*HesTKO* mutants have a statistically significant increase in Pax2+ cells. There are not more Pax2+Pax6+ double labeled cells. Only this particular genotype has an increase in Pax2+ single labeled cells.
5a) Page 11, line 20-25. There seems to be inconsistency between result description and image data of Fig. 5A-G, and histogram Fig. 5O. Authors mentioned that a modest loss of pH3+ cell fraction in Chx10-Cre; Hes1/3/5 cTKO but not in Rax-Cre; Hes1/3/5 cTKO. However, Fig. 5D indicates severe reduction of pH3+ cell fraction in Rax-Cre; Hes1/3/5/ cTKO, which is similar to reduction of pH3+ cell fraction in Rex-Cre; Rbpj (Fig. 5B), but histogram data is different (Fig. 5O). Furthermore, pH3+ cell fraction is severely reduced in Chx10-Cre; ROSA(dn-Maml-GFP) (Fig. 5F) and modestly reduced in Chx10-Cre; Hes1/3/5 cTKO (Fig. 5G). However, pH3+ cell fraction seems to be normal in Chx10-Cre; Rbpj (Fig. 5E). These Chx10-Cre image data do not match the histogram of Fig. 5O. Please check their situation.

Response: Images in old Figs 5-8 were normalized using area measurements, see methods and above comments (note: old Figs 6&7 were combined into new Fig 6). One-way ANOVA with pairwise comparisons for each mutant genotype compared to control were calculated using Prism. All genotypes except two have a statistically significant loss of M phase cells and we discuss possibilities for this outcome (Fig 5O). A normalization method for the sampled area is an essential component of these studies since morphologic differences are apparent for particular genotypes. The quantitative data are consistent with our original conclusions.

5b) Fig. 5H-N, P: I wonder if the stage E13 is appropriate to evaluate cell death and survival because optic cup already becomes smaller in Rax-Cre; Rbpj, Hes1/3/5 cTKO, or ROSA(dn-MAML-GFP) than in wild-type control. I suggest the authors examine more earlier stage.

Response: While an earlier effect is possible, we only observed size differences in a subset of the genotypes. Thus, E13 serves as a critical timepoint to examine early developmental phenotypes across the totality of our mutant conditions. It is also first age when the ONH is fully formed.

5c) Page 12, line 19-20, "all other mutants (Chx10-Cre; Rbpj, and Chx10-Cre; ROSA(dn-MAML-GFP) were unaffected (Fig. 6EF, LM, ST)": It is likely that atoh7 expressing cells are mildly decreased and neuronal marker, Tubb3 and Rbpms-expressing cells are increased in Chx10-Cre; Rbpj, and Chx10-Cre; ROSA(dn-MAML-GFP). I requested the authors to evaluate the fraction of these markers in retinal area statistically in all the cases.

Response: As described above, we quantified Atoh7 and Rbpms nuclear expression by immunohistochemistry. We do not believe that Tubb3+ cells can be reliably quantified. Nonetheless, it is useful to qualitatively show the extent of excess neuron formation. Importantly, we observed that it is not the Atoh7 status that matters for RGC formation, rather it is the Otx2 expression status. This is in good agreement with single cell-RNA transcriptomics data from Wu et al 2021 showing that *Atoh7* mRNA in all early transitional RPCs remains fairly constant and its loss does not block the formation of early RGC cell states (13). By contrast Otx2 fluctuates but remains expressed in transitional RPCs that progress to photoreceptor lineages.

6a) Page 7, line 19 "Ectopic blood vessels protruded from the ONH (Fig. 1K, 1L)": It is difficult to see blood vessel structures in these panels (Fig. 1I-L). Please show some molecular marker of blood vessels to confirm how blood vessel is organized in Hes1/3/5 cTKO.

Response: These vascular structures are highly conspicuous by morphology in the H&E insets. Nonetheless, we used adjacent P21 sections to immunolabel for Endomuscin (14) and Tubb3 antibodies. This colabeling confirms the morphology and position of ectopic blood vessels in the abnormal tissue masses in Chx10-Cre;*HesTKO* mutant eyes. Ectopic tissue contains only rare Tubb3+ cells or cell processes suggesting it is overwhelmingly nonneural. All P21 data were moved to a new Suppl Fig 2. A full detailing of vascular phenotypes is beyond the scope of this manuscript and, interestingly, would be potentially attributable to non-autonomous effects of perturbing the *Hes* genes in the adjacent retina.

6b) Fig. 5: Increase of pH3 fraction indicates several possibilities, for example (1) increased fraction of mitotic cells due to precocious neurogenesis, (2) increased fraction of mitotic cells due to activated cell proliferation of retinal progenitor cells, (3) increased cell-cycle arrest in M phase due to some stress response of progenitor cells. So, I suggest the authors to examine (1) BrdU percentage of retinal section area, (2) the percentage of pH3+ cells in PCNA+ retinal cells.

Response: The data listed in Suppl Table 1 presents a unified picture that disrupting Notch signaling reduced proliferation. This paradigm extends to other model organisms (*e.g.*, *Drosophila*, chick, frog, zebrafish and even to nonneural tissues). We included the phospho-histone H3 staining so readers would see how the six mutants evaluated in this study align with this paradigm, providing confidence for the novel findings in other figures. A full evaluation of cell cycle kinetics is interesting, but beyond the scope and focus of this manuscript.

6c) Fig. 5: It is better that cell death fraction will be evaluated by TUNEL and labeling with anti-activated caspase 3 antibody.

Response: We disagree. The DNA repair enzyme PARP is inactivated upon cleavage by activated caspase 3. There are currently ~3,600 citations that use it as a marker of apoptosis. PARP also has a separate and very specific role in maintaining the integrity of sperm DNA. This antibody works on all metazoans and is amenable to many tissue preparations and fixatives, making it easy to use, robust and quantifiable.

7a) Please show red channel (Hes1) image in Fig1BC.

Response: This was added to Revised Fig 1 (Fig 1A).

7b) Fig. 1DH should be shown in neighbor. Fig. 1H should be assigned as Fig. 1E.

Response: The new Fig 1 layout addresses this point.

7c) Fig. S2D, F, H, J: Please show GFP green channel as well. Otherwise, it is difficult to see non-overlapping expression in optic stalk area.

Response: In the revision, this is Suppl Fig 3. Chx-10-Cre is not expressed by ONH-OS cells (1). The green and fuchsia overlap (coexpression) in RPCs is white, we feel this is fairly clear. If needed, all readers can turn on and off the green channel in the final PDF version of this figure to compare GFP with Hes1 expression for those panels.

7d) Fig. 9B: It is better to show Rax-Cre: Hes1/3/5 TKO rather than Rax-Cre: Hes1 cKO. 
7e) Fig. 9B: Lettering "Rbpj mutant" should be revised as "Rax-Cre: Rbpj KO".

Response: Fig 9B was removed so these terms are now irrelevant. Our models are presented in new Fig 8.

Significance: The senior author of this manuscript, Dr. Nadean Brown, is an expert scientist who has investigate the role of Notch signaling pathway in vertebrate ocular tissue, including the neural retina and lens. In general, Notch signaling pathway consists of signaling stream from the interaction of Delta and Notch, Notch receptor activation by proteolytic cleavage, translocation of Notch intracellular domain (NICD) into nucleus, formation of transcription factor complex consisting of NICD/Rbpj/Maml, to the transcriptional activation of Notch target genes, Hes family transcription factors. Finally, Hes suppresses neurogenic program and maintain a pool of neural progenitor cells. Therefore, Notch is a key factor to regulate the balance between neurogenesis and progenitor proliferation. In this manuscript, the authors investigated retinal phenotypes in the knockout mice of different Notch signaling components, including Rbpj, Maml, and Hes. They found that functions of these three factors are not always equal in retinal cell differentiation; rather, they specifically regulate a particular step of retinal development. The authors propose the possibility that each of Notch signaling components may be modified by other signaling pathways and achieve some new roles beyond the conventional frame of classic Notch signaling pathway. In this point, this work has a potential to provide a new conceptual advance in the field of developmental and cell biology.

We fully agree this work is a significant advance for the fields of developmental and cell biology. Our findings provide new information and stimulate fresh ideas for anyone working on signal transduction and signal integration.

References cited:

1. Bosze et al., 2020 Journal of Neuroscience Vol 40:1501-13; Bosze et al. 2021 Dev Biol Vol 472:18-29.

2. Han et al., 2023 Development Vol 150 dev201408.

3. Kopan and Ilagan, 2004 Nat Rev Cell Biol. Vol 5:499-504

4. Hirata et al., 2002 Science Vol 298:840-3

5. Friedmann and Kovall, 2010 Protein Sci. Vol 19:34-46

6. Ong et al., 2006 JBC Voll24:5106-19

7. Wall et al., 2009 J Cell Biol. Vo 184: 101-12.

8. Javed et al., 2023 Development Vol 150:dev200436

9. Matuda and Cepko 2007 PNAS Vol 104: 1027-1032

10. Ohtsuka et al., 2006 Mol. Cell Neurosci. Vol 31:109-22

11. Zheng et al., 2009 Molecular Brain Vol 2:38

12. Smith et al., 2017 Journal of Neuroscience Vol 37:7975-93.

13. Wu et al., 2021 Nature Communications Vol 12:1465: doi 10.1038/s41467-021-21704-4

14. Saint-Geniez et al., 2009 IOVS Vol 50: 311-21.
