# Supplementary material for: Notch pathway mutants do not equivalently perturb mouse embryonic retinal development
Source: PLoS Genet. 2023 Sep 26;19(9):e1010928. doi: 10.1371/journal.pgen.1010928 (PMC10522021; doi:10.1371/journal.pgen.1010928)
Supplement: Peer review history

# Author comment: Author response to previous submission

**DOI:** 10.1371/journal.pgen.1010928.r001

11 Jul 2023

Attachment Submitted filename: 133380_1_rebuttal_2729441_rxq268.docx Click here for additional data file.

# Peer review: Decision Letter 0

**DOI:** 10.1371/journal.pgen.1010928.r002

**Authors:** Gregory S. Barsh, Seth Blackshaw

16 Aug 2023

Dear Dr Brown,

Thank you very much for submitting your Research Article entitled 'Notch pathway mutants do not equivalently perturb mouse embryonic retinal development' to PLOS Genetics.

The manuscript was fully evaluated at the editorial level and by independent peer reviewers. The reviewers appreciated the attention to an important problem, but raised some substantial concerns about the current manuscript. Based on the reviews, we will not be able to accept this version of the manuscript, but we would be willing to review a much-revised version. We cannot, of course, promise publication at that time.

Should you decide to revise the manuscript for further consideration here, your revisions should address the specific points made by each reviewer. We will also require a detailed list of your responses to the review comments and a description of the changes you have made in the manuscript.

If you decide to revise the manuscript for further consideration at PLOS Genetics, please aim to resubmit within the next 60 days, unless it will take extra time to address the concerns of the reviewers, in which case we would appreciate an expected resubmission date by email to plosgenetics@plos.org.

If present, accompanying reviewer attachments are included with this email; please notify the journal office if any appear to be missing. They will also be available for download from the link below. You can use this link to log into the system when you are ready to submit a revised version, having first consulted our Submission Checklist.

To enhance the reproducibility of your results, we recommend that you deposit your laboratory protocols in protocols.io, where a protocol can be assigned its own identifier (DOI) such that it can be cited independently in the future. Additionally, PLOS ONE offers an option to publish peer-reviewed clinical study protocols. Read more information on sharing protocols at https://plos.org/protocols?utm_medium=editorial-email&utm_source=authorletters&utm_campaign=protocols

Please be aware that our data availability policy requires that all numerical data underlying graphs or summary statistics are included with the submission, and you will need to provide this upon resubmission if not already present. In addition, we do not permit the inclusion of phrases such as "data not shown" or "unpublished results" in manuscripts. All points should be backed up by data provided with the submission.

While revising your submission, please upload your figure files to the Preflight Analysis and Conversion Engine (PACE) digital diagnostic tool.  PACE helps ensure that figures meet PLOS requirements. To use PACE, you must first register as a user. Then, login and navigate to the UPLOAD tab, where you will find detailed instructions on how to use the tool. If you encounter any issues or have any questions when using PACE, please email us at figures@plos.org.

PLOS has incorporated Similarity Check, powered by iThenticate, into its journal-wide submission system in order to screen submitted content for originality before publication. Each PLOS journal undertakes screening on a proportion of submitted articles. You will be contacted if needed following the screening process.

To resubmit, use the link below and 'Revise Submission' in the 'Submissions Needing Revision' folder.

We are sorry that we cannot be more positive about your manuscript at this stage. Please do not hesitate to contact us if you have any concerns or questions.

Yours sincerely,

Seth Blackshaw

Guest Editor

PLOS Genetics

Gregory Barsh

Editor-in-Chief

PLOS Genetics

This manuscript has now been reviewed by three experts in the field. All consider the study to be both important for the field of early development and well-executed overall. However, all three also agree that the data presented needs quantifying, along with appropriate statistical analysis. Where practical (i.e. with <3 months additional work), these should be included. All reviewers likewise agree that the title should be changed to better reflect the analysis of the differential contributions of Rbpj and Hes signaling, which is the core of the study. While the manuscript is generally well-written, some additional background for readers not working directly on the (highly complex) details of Notch signaling, and well as some reorganization of the figures to better reflect the narrative flow, would also be desirable.

# Editor report: Acceptance letter

**DOI:** 10.1371/journal.pgen.1010928.r003

**Authors:** Gregory S. Barsh, Seth Blackshaw

12 Sep 2023

PGENETICS-D-23-00774 

Notch pathway mutants do not equivalently perturb mouse embryonic retinal development 

Dear Dr Brown, 

We are pleased to inform you that your manuscript entitled "Notch pathway mutants do not equivalently perturb mouse embryonic retinal development" has been formally accepted for publication in PLOS Genetics! Your manuscript is now with our production department and you will be notified of the publication date in due course.

The corresponding author will soon be receiving a typeset proof for review, to ensure errors have not been introduced during production. Please review the PDF proof of your manuscript carefully, as this is the last chance to correct any errors. Please note that major changes, or those which affect the scientific understanding of the work, will likely cause delays to the publication date of your manuscript. 

Soon after your final files are uploaded, unless you have opted out or your manuscript is a front-matter piece, the early version of your manuscript will be published online. The date of the early version will be your article's publication date. The final article will be published to the same URL, and all versions of the paper will be accessible to readers.

Thank you again for supporting PLOS Genetics and open-access publishing. We are looking forward to publishing your work! 

With kind regards,

Livia Kovacs

PLOS Genetics

On behalf of:

The PLOS Genetics Team

Carlyle House, Carlyle Road, Cambridge CB4 3DN | United Kingdom

plosgenetics@plos.org | +44 (0) 1223-442823

plosgenetics.org | Twitter: @PLOSGenetics
